# Pollination ecology in the tropical Andes: moving towards a cross‐scale approach

**DOI:** 10.1111/brv.70049

**Published:** 2025-07-15

**Authors:** Cristina Rueda‐Uribe, Alexander Chautá, Tamsin L. Woodman, Eloisa Lasso, Roxibell C. Pelayo, Laura Milena Manrique‐Garzón, Marcia C. Muñoz, Rebekka Allgayer, Tia‐Lynn Ashman, Greta Bocedi, David F.R.P. Burslem, Pedro A. Camargo‐Martínez, María Ángela Echeverry‐Galvis, Catalina González‐Arango, Cecile Gubry‐Rangin, Lesley T. Lancaster, Kara K.S. Layton, Fabio Manfredini, Carlos Martel, Lia Montti, Alexander S.T. Papadopulos, Robert A. Raguso, Jonathan Ready, Alejandro Rico‐Guevara, Camila Rocabado, Justin M. J. Travis

**Affiliations:** ^1^ School of Biological Sciences, University of Aberdeen Zoology Building Tillydrone Ave. Aberdeen AB24 2TZ UK; ^2^ Department of Entomology Cornell University Comstock Hall, 129 Garden Ave Ithaca NY 14853 USA; ^3^ School of Geosciences, University of Edinburgh Drummond Street Edinburgh EH8 9XP UK; ^4^ Estación Científica Coiba AIP Ciudad del Saber, Clayton, Calle Gustavo Lara #145B Panamá 07144 República de Panamá; ^5^ Smithsonian Tropical Research Institute Roosevelt Ave, Tupper BLDG 401 Ancón 07098 República de Panamá; ^6^ Instituto de Ciencias Ambientales y Ecológicas, Facultad de Ciencias Universidad de Los Andes, Avenida Alberto Carnevali Núcleo pedro Rincón Gutiérrez, La Hechicera Mérida 5101 Venezuela; ^7^ Departamento de Ciencias Biológicas Universidad de los Andes Edificio J, Carrera 1 # 18A‐ 12 Bogotá 111711 Colombia; ^8^ Programa de Biología Universidad de La Salle Calle 11 # 1‐02 Bogotá Colombia; ^9^ Department of Biological Sciences University of Pittsburgh Pittsburgh PA 15260 USA; ^10^ Parque Nacional Natural Chingaza La Calera Colombia; ^11^ Departamento de Ecología y Territorio, Facultad de Estudios Ambientales y Rurales Pontificia Universidad Javeriana Trans 4 # 42‐00, Edificio 67, piso 8 Bogotá 110231 Colombia; ^12^ Department of Biology University of Toronto Mississauga Davis Building 3359 Mississauga Rd Mississauga L5L 1C6 Canada; ^13^ Jodrell Laboratory, Royal Botanic Gardens Kew Richmond TW9 3DS UK; ^14^ Instituto de Ciencias Ómicas y Biotecnología Aplicada Pontificia Universidad Católica del Perú San Miguel Lima 15088 Peru; ^15^ Instituto de Investigaciones Marinas y Costeras‐CONICET, Instituto de Geología de Costas y del Cuaternario‐CIC Facultad de Ciencias Exactas y Naturales Universidad Nacional de Mar del Plata, Funes 3350 Mar del Plata 7600 Argentina; ^16^ Molecular Ecology and Evolution group School of Environmental and Natural Sciences, Environment Centre Wales, Bangor University Bangor LL57 2UR UK; ^17^ Department of Neurobiology and Behavior Cornell University Seeley G Mudd Hall 215 Tower Road Ithaca NY 14853 USA; ^18^ Centro de Estudos Avançados da Biodiversidade Universidade Federal do Pará R. Augusto Corrêa, 1, Guamá Belém PA 66075‐110 Brazil; ^19^ Department of Biology University of Washington 3747 W Stevens Way NE Seattle WA 98195 USA; ^20^ Burke Museum of Natural History and Culture University of Washington 4303 Memorial Way NE Seattle WA 98195 USA

**Keywords:** biotic interactions, ecosystem functioning, ecological monitoring, plant–pollinator networks, pollinators, South America

## Abstract

Plant–pollinator interactions structure ecological communities and represent a key component of ecosystem functioning. Pollination networks are expected to be more diverse and specialised in the tropics, but pollination ecology in these regions has been understudied in comparison to other areas. We reviewed research on pollination in the tropical Andes, one of the major biodiversity hotspots on Earth, where the uplift of mountains and past climate have resulted in spatiotemporally distinct species interactions. We found 1010 scientific articles on pollination in the Andes, of which 473 included or were carried out in tropical regions. The number of publications on pollination ecology in the tropical Andes has increased exponentially, with Colombia having the most articles, followed by Ecuador and Peru, and with Bolivia and Venezuela having notably fewer studies. More research has been carried out in humid montane forests and agricultural landscapes, and it has predominantly focused on describing diversity of species and interactions while neglecting analyses on the resilience and adaptability of pollinating systems, even though the Andean region is particularly susceptible to the effects of climate change and continues to undergo land conversion and degradation. Remarkably few studies have incorporated local knowledge, thus ignoring connections to human livelihoods and communities. A phytocentric perspective has been predominant, with fewer studies focusing directly on pollinators and a notable lack of articles with a holistic approach to the study of pollination across taxonomic groups at the community or ecosystem level. We propose that future research adopts a cross‐scale approach that considers the complexity of the ecological contexts in which plant–pollinator interactions occur, and incorporates long‐term monitoring with broader multilayer networks and molecular tools, experiments focused on ecophysiology and behaviour, animal telemetry, process‐modelling approaches and participatory science. A stronger field driven by interdisciplinary collaborations will contribute to knowledge about pollination at a global scale, as well as increase our understanding of the diversity and resilience of pollination interactions in this region, thus improving our capacity to predict and avoid ecosystem collapses.

## I. INTRODUCTION

Pollinators shape the reproduction, gene flow and population dynamics of plants, while plants provide food resources that are vital for the survival, abundance and diversity of pollinators. Although plants can also reproduce without the aid of pollinators (i.e. through abiotic means or self‐pollination), most angiosperms depend on animals for pollen transfer and increased fecundity (Ollerton, Winfree & Tarrant, 2011; Rodger *et al*., 2021; Stephens *et al*., 2023). The interactions between pollinators and pollinated plants form networks that are key for ecosystem functioning (Schleuning, Fründ & García, 2015) and, through evolutionary time, have been a major contributor to patterns of terrestrial biodiversity (Grimaldi, 1999; van der Niet & Johnson, 2012). The interdependence of species in plant–pollinator interactions is shaped by ecological and evolutionary contexts, with complex patterns of coevolutionary mosaics (Thompson, 2005) and variation in their robustness and resilience to change (Kaiser‐Bunbury *et al*., 2010). With ongoing global changes, the importance of animal pollination as an underlying ecosystem service for food production, ecosystem restoration, and human welfare has been recognised as a high priority (Potts *et al*., 2016b). Plant–pollinator interactions have therefore been the object of extensive study, with efforts made to increase our understanding of the drivers and consequences of such interactions, and aims ranging from advancing conceptual developments to addressing more applied goals such as sustainable agriculture (Mayer *et al*., 2011; Knight *et al*., 2018; Tong, Wu & Huang, 2021).

Still, pollination interactions in tropical regions remain largely understudied (Vizentin‐Bugoni *et al*., 2018), even though they exhibit remarkable variation and distinctiveness, have high species diversity and endemism, and are particularly vulnerable to ongoing anthropogenic changes. Within the tropics, the Andes Mountains stand out as one of the world's biodiversity hotspots (Myers *et al*., 2000). It is estimated that floral richness in the Andes exceeds 28,000 known species (Pérez‐Escobar *et al*., 2022), with many more yet to be described (Ondo *et al*., 2023). Similarly, several animal lineages in this region, including potential pollinating species, also reach record levels of diversity (Raven *et al*., 2020). Plants and animals in the Andes are also highly endemic, with many species having small ranges and occupying narrow niches (Lamoreux *et al*., 2006; Sandel *et al*., 2020), which makes them especially vulnerable to anthropogenic pressures because local extirpations may rapidly escalate to species extinctions when species ranges are small (Chichorro, Juslén & Cardoso, 2019; Staude, Navarro & Pereira, 2020; Manes *et al*., 2021; Trew & Mclean, 2021). Furthermore, endemic and threatened species in this region are insufficiently protected (Bax & Francesconi, 2019).

There is thus an urgent need for further investigation into plant–pollinator dynamics in tropical Andean ecosystems. Additional research on pollination ecology in this region will enhance management and restoration strategies, deepen our understanding of local Andean landscape complexities and contribute to the knowledge of pollination ecology globally. In this review, we initially describe the region, its major ongoing and future threats, and its uniqueness in terms of pollination systems (Section I). We then review current knowledge gaps about pollination ecology in the tropical Andes (Section II), propose future avenues that are critical for advancing research and informing conservation efforts (Section III), and outline major conclusions (Section IV).

### (1) Description of the tropical Andes

**Fig. 1:**
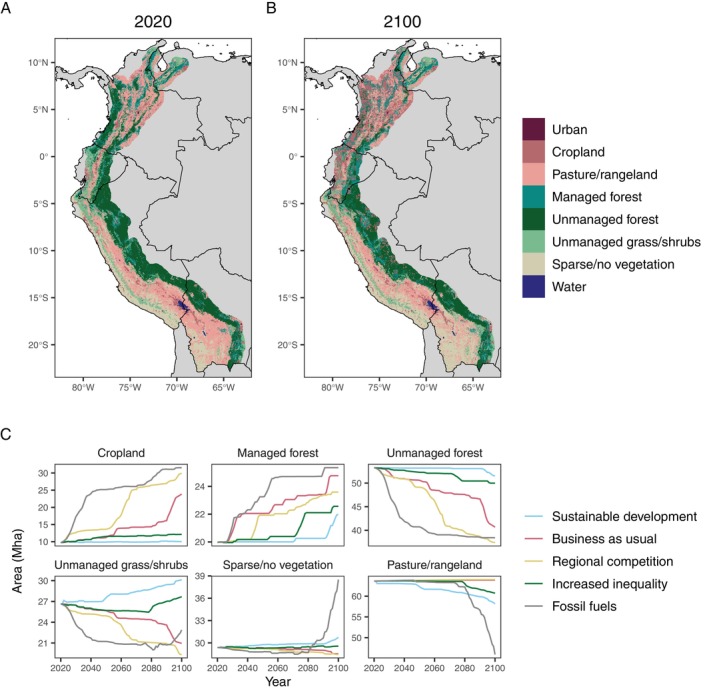
Current and future land cover projections for the tropical Andes region. For this literature review, the tropical Andes region was defined as occurring north of the Tropic of Capricorn (23.4394° S), excluding areas in Chile and Argentina. (A) Current land cover for the region in 2020 at 1 km spatial resolution from the HILDA+ version 2b data set (Winkler *et al*., 2025). (B) Future land cover projections for 2100 under SSP5‐RCP8.5 (socioeconomic scenario of growth fuelled by fossil fuel consumption). (C) Projected areas of six land use and land cover classes from 2020 to 2100 under five socioeconomic scenarios (SSP1‐RCP2.6, sustainable development; SSP2‐RCP4.5, business as usual, SSP3‐RCP7.0, regional competition; SSP4‐RCP6.0, increased inequality, and SSP5‐RCP8.5, fossil fuels). Note that ‘Unmanaged grass/shrubs’ is equivalent to ‘Unmanaged grass/shrublands’ in the HILDA+ data set. The map for A and B was extracted from the GMBA Mountain Inventory (Snethlage *et al*., 2022a,b) and we added a 50 km buffer to include lowland areas that are still under Andean influence (e.g. inter‐Andean valleys and foothills). See Fig. S1 for projections by country.

The tropical Andes Mountains occur broadly between 10° N and 23.4394° S (Tropic of Capricorn) on the western part of the South American continent. The region defined as the tropical Andes in our study includes the mountainous formations of Andean origin in Venezuela, Colombia, Ecuador, Peru and Bolivia (Fig. 1). The Andes Mountain Range is also divided geologically into North, Central and South regions according to the position of the underlying tectonic plates, but we refer herein to the tropical and subtropical ecosystems north of the tropic of Capricorn as the ‘tropical Andes’ for simplicity and due to its ecological significance (Luteyn & Churchill, 2000).

The ecosystems of the tropical Andes differ from those of the temperate Andes in that temperatures in tropical regions do not have marked seasonal fluctuations and growing seasons can span the entire year, although rainfall is highly variable (Young *et al*., 2007). Ranging from around 500 m above sea level (a.s.l.) up to summits over 6000 m a.s.l. (Josse *et al*., 2011), the tropical Andes hosts a variety of ecosystems including humid and dry forests, shrublands, and open grasslands that change drastically in abiotic and biotic conditions along elevation gradients (Cuatrecasas, 1958; van der Hammen, 1984; Graham, 2009; Cleef, 2013). In fact, voyages throughout the Andes and observations of vegetation zonation along the slopes of Mt. Chimborazo in Ecuador inspired Alexander von Humboldt to conceptualise habitat stratification and turnover in elevation gradients as a foundational concept in biogeography and a major driver of biodiversity (Rahbek *et al*., 2019). Diversification across this region has been repeatedly linked to the uplift of the Andes mountains (Hughes & Eastwood, 2006; Antonelli *et al*., 2009; Chaves, Weir & Smith, 2011; Pérez‐Escobar *et al*., 2017) and is further influenced by the complexity of climatic history, environmental change and biotic interactions (Luebert & Weigend, 2014; Cadena, Pedraza & Brumfield, 2016; Lagomarsino *et al*., 2016; Flantua *et al*., 2019; Coelho *et al*., 2023).

Regardless of the ecological and evolutionary mechanisms driving species richness (Graham *et al*., 2014), it is undeniable that the tropical Andes is one of the most diverse regions on the planet (Myers *et al*., 2000). Different life forms are distributed from lowland habitats in inter‐Andean valleys and foothills to ecosystems of paramo or puna that are just below the rocky or snow‐covered mountain tops. Andean cloud forests at mid elevations (~1000–2000 m a.s.l.) are exceptionally diverse across taxonomic groups (Kessler *et al*., 2001; Kattan *et al*., 2004; Hutter, Lambert & Weins, 2017), but even elevations over 3000 and 4000 m a.s.l., exhibit adaptive radiations in some plant taxa [e.g. *Lupinus* (Hughes & Eastwood, 2006); multiple clades in the paramo (Madriñán, Cortés & Richardson, 2013); *Espeletia* (Pouchon *et al*., 2018)] and these ecosystems have a higher species richness than other tropical alpine regions, such as those in East Africa and New Guinea (Sklenář, Hedberg & Cleef, 2014).

### (2) Threats to tropical Andean ecosystems

The tropical Andes face the same major challenges that threaten ecosystems at a global scale, but this region is especially vulnerable to climate change and has been subject to intense landscape degradation and fragmentation (Young, Young & Josse, 2011; Armenteras *et al*., 2017). With increasing temperatures and changing precipitation regimes, the ranges of montane plants and animals are predicted to shift upwards in elevation (Larsen *et al*., 2011; Buytaert, Cuesta‐Camacho & Tobón, 2011; Bender *et al*., 2019; Tovar *et al*., 2022). Expected shifts in patterns also include increased tree cover in areas that are currently open ecosystems above the treeline (Morueta‐Holme *et al*., 2015; Arzac *et al*., 2019), contraction in species' ranges (Báez *et al*., 2016), and local extinctions on mountain summits (Forero‐Medina, Joppa & Pimm, 2011; Freeman *et al*., 2018). Although rainfall may increase in some areas, such as in forests located on the Pacific slopes in Ecuador and Colombia, the hydrological integrity of Andean ecosystems is threatened by reduced water supply from glaciers (Bradley *et al*., 2006), and changes in moisture transport from the Amazon basin (Arias *et al*., 2023) leading to longer dry seasons that might compromise biological stability and the overall health of ecosystems (Cresso *et al*., 2020). Temperature has increased and is predicted to produce warmer conditions, particularly at higher elevations, meaning that alpine ecosystems such as paramo and puna may be at higher risk (Vuille *et al*., 2015). In these vulnerable ecosystems, research has demonstrated that a shortage of water and increased temperatures cause physiological stress for plants, even though some species may be more tolerant than others (Ayarza‐Páez, Garzón‐López & Lasso, 2022), making whole‐ecosystem responses difficult to determine. Similarly, different thermal tolerances have been found for bumblebees and stingless bees across elevation gradients, but species are thermally adapted to local climates and might be affected by changes in humidity with increased warming (González *et al*., 2022a,b). As in other regions, plant–pollinator interactions are directly affected by climate change through possible changes in pollinator behaviour, plant or pollinator physiology, morphology or abundance, and spatiotemporal mismatches (Aguirre *et al*., 2011; Settele, Bishop & Potts, 2016; Adedoja, Kehinde & Samways, 2020; Sonne, Maruyama & Martín González, 2022b), impacting not only natural ecosystems but also related services to humans. For example, climate projections predict substantial reductions in species distributions of stingless bees used in meliponiculture in Colombia, threatening agricultural production and human livelihoods (González *et al*., 2021).

In addition, the Andes mountains have been historically transformed by human presence for thousands of years, long before the colonial period (Baied & Wheeler, 1993; Goldberg, Mychajliw & Hadly, 2016). Through time, wild landscapes have been impacted by agriculture, urban settlement, grazing, and mining (Etter, McAlpine & Possingham, 2008; Armenteras *et al*., 2017; Rolando *et al*., 2017). Major cities and towns have expanded the human footprint in the region (Correa Ayram *et al*., 2020), and the influx and movement of human communities have brought about the introduction of exotic species, profoundly altering natural ecosystems (González *et al*., 2024). Today, the Andes are still under pressure from growing human populations and intensified production systems that cause land degradation and fragmentation, with several Andean ecosystems, including both humid and dry forests, already having lost more than 50% of their extent since preindustrial times (Comer *et al*., 2022).

To assess how the loss of natural ecosystems is expected to continue in the tropical Andes under different scenarios of socioeconomic development, we mapped current and future land cover projections for the region (Fig. 1) using the map for the Andes Mountains extracted from the GMBA Mountain Inventory (Snethlage *et al*., 2022a,b). We used 1 km spatial resolution from the HILDA+ version 2b data set (Winkler *et al*., 2025) and downscaled future land use and land cover change projections from LandSyMM (Rabin *et al*., 2020) using the LandScaleR downscaling algorithm (Woodman *et al*., 2023; T. L. Woodman, B. Arendarczyk, K. Winkler, R. C. Henry, F. Eigenbrod, D. F. R. P. Burslem, P. Alexander & J. M. J. Travis, in preparation; see online Supporting Information, Appendix S1 for methods). We found that the expansion of cropland and managed forest cover in the tropical Andean region, for example, is projected to drive the loss of an additional 15 Mha of unmanaged forest and 4 Mha of unmanaged grass/shrubland areas between 2020 and 2100 under scenario SSP5‐RCP8.5, where future economic growth is sustained by fossil fuel consumption. Natural unmanaged forest areas are predicted to decrease by 2 Mha even under the scenario of sustainable development (SSP1‐RCP2.6; Fig. 1C). This pattern suggests that forests will be threatened by future land cover change if no mitigating actions are taken across the region. Substantial losses of unmanaged forests are expected to occur particularly in Colombia, where 7.67 Mha of unmanaged forest are projected to be lost by 2100 with business as usual (SSP2‐RCP4.5) in our defined Andean region (Fig. S1). Under the same scenario, the areas of unmanaged forest that will be lost by 2100 in the region for each country are: 2.38 MHa in Ecuador, 2.27 MHa in Peru, 0.16 MHa in Bolivia, and 0.11 MHa in Venezuela (Fig. S1). Habitat degradation and loss cause local extinctions that cascade through pollination networks, directly and indirectly affecting plants and pollinators and altering ecosystem stability as well as pollination services to crops (Pires *et al*., 2020; Bascompte & Scheffer, 2023). In short, due to their outstanding biodiversity and current and future threats, the tropical Andean mountains remain a global conservation priority, and the threats to these ecosystems constitute a threat to the communities of plants and pollinators within them (e.g. Martins *et al*., 2015).

### (3) Unique plant–pollinator interactions

**Fig. 2:**
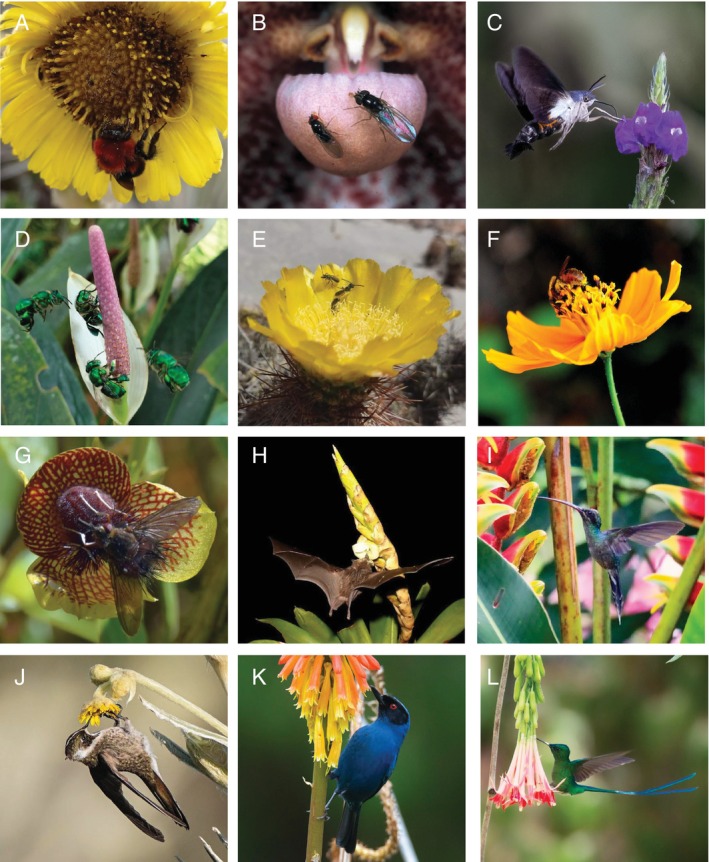
Photographs showcasing examples of plant–pollinator interactions in the tropical Andes. (A) *Bombus rubicundus*, a pollinator that is an important hub of interactions both within and between modules in plant–pollinator networks at high elevations, visiting rosetted plants (*Espeletia grandiflora*) endemic to paramos. (B) Two flies (left, *Hirtodrosophila* sp. and right, *Zygothrica antedispar*) attracted to the mushroom‐smelling orchid *Dracula laffleurii*. (C) Diurnal hawkmoth visiting flowers of *Stachytarpheta* cf. *cayennensis*, an exotic verbena flower popular in ornamental gardens. (D) Orchid bees of the genus *Euglossa* visiting flowers of *Anthurium antioquiense*, a plant endemic to the Central Cordillera of Colombia. (E) Solitary male bees of the genus *Caenohalictus* visiting a flower of the cactus *Cumulopuntia sphaerica* in mountainous desert shrubland, where these bees are frequent pollinators of cacti but have been largely understudied. (F) Stingless bee *Melipona* sp. in a sanctuary dedicated to meliponiculture, visiting a native aster, *Cosmos* sp., that is commonly used to attract insect pollinators to gardens and crops. (G) A male fly of *Eudejeania* sp. pseudocopulating with a flower of the sexually deceptive orchid *Telipogon salinasiae*. (H) *Anoura fistulata*, the nectar bat with the longest tongue relative to its body recorded for any mammal, drinking from the flowers of bromeliad *Werauhia gladioliflora*. (I) *Phaethornis guy*, a hermit hummingbird with a long, curved bill visiting *Heliconia* sp. by hovering. (J) *Oxypogon guerinii*, a hummingbird with a short, straight bill visiting *Espeletia grandiflora* by clinging to the flowers. (K) Nectar‐robbing by *Diglossa cyanea* through holes it pierces in the base of the corolla of an exotic *Kniphofia* sp. (L) Nectar‐robbing by *Aglaiocercus kingii* from *Fuchsia* sp., using holes previously made by flowerpiercers. Photographs by Laura Milena Manrique‐Garzón (A), Lorena Endara (B), Diego Emerson Torres (C), Daniel Salazar Rios (D), Yeison Calizaya Melo (E), José Isidro Vargas (F), Carlos Martel (G), Nathan Muchhala (H), and Pedro A. Camargo‐Martínez (I–L).

The rugged topography, together with the dynamic geological and climatic history of the Andes, results in a highly heterogeneous landscape that shelters high endemism and diversity (Llambí & Rada, 2019), and thus the potential for unique plant–pollinator interactions with a striking richness of adaptations and interactions (Fig. 2), with new descriptions of species and pollination systems, even in recent years (e.g. Kawakita *et al*., 2019; Martel, Francke & Ayasse, 2019). Fluctuations in past climate have repeatedly shifted vegetation bands along elevational gradients of mountain slopes, a phenomenon coined ‘flickering connectivity’ (Flantua *et al*., 2019) that results in iterative periods of isolation, differentiation and speciation (Hughes & Eastwood, 2006; Rahbek *et al*., 2019). Importantly, pollination interactions themselves play a central role in driving diversification. Several mechanisms that contribute to diversification related to plant–pollinator interactions have been described using examples from the Andean region, including floral adaptation to pollinators (Muchhala & Potts, 2007; Smith, Ané & Baum, 2008a), changes in plant reproductive strategies (Lagomarsino *et al*., 2016), and coevolutionary dynamics between interacting partners or guilds (Abrahamczyk, Souto‐Vilarós & Renner, 2014; Abrahamczyk, Poretschkin & Renner, 2017; Ibañez *et al*., 2019). The wealth of functional variation in tropical Andean plant clades in particular has stimulated research about transitions in adaptation to pollinators through the study of pollination syndromes (e.g. Lagomarsino *et al*., 2016, 2017; Smith & Kriebel, 2018). Pollination syndromes are suites of floral traits related to attracting certain functional groups of pollinators (*sensu* Fenster *et al*., 2004). Shifts in pollination syndromes through evolutionary time can be detected by mapping floral traits on phylogenetic trees, and these shifts can be driven by selection to reduce the extent of interspecific competition among plants (Muchhala, Johnsen & Smith, 2014; Cuartas‐Hernández *et al*., 2019) or expand the breadth of potential pollinators and increase pollination efficiency (Dellinger *et al*., 2019).

Examples of extreme morphological and ecological specialisation also exist, despite the risks of relying on just a single or a few interaction partners. Such is the case for two plants from different families (*Centropogon nigricans*, Campanulaceae and *Marcgravia williamsii*, Marcgraviaceae) in the Ecuadorian cloud forests that have a single recorded floral visitor: *Anoura fistulata*, the tube‐lipped nectar bat (Muchhala *et al*., 2024). This bat's tongue is so long that it is the only one capable of reaching the nectar deep within the flowers of these plants. Measuring 150% of the bat's body length, the tongue retracts into a glossal tube in the bat's thoracic cavity (Muchhala, 2006a). The risk inherent in increased specialisation is relying on a single or reduced set of interaction partners, which can lead to coevolutionary escalation (Thompson, 1994) and an increased vulnerability to disturbance (Bascompte & Scheffer, 2023). Interestingly, in harsh habitats with lower pollinator richness and abundance, differences in mating systems arise, with potentially lower levels of outcrossing due to the predominance of generalised pollination (Cristóbal‐Pérez *et al*., 2024; Manrique‐Garzón *et al*., 2025), abiotic reproductive strategies (e.g. wind pollination in high‐elevation *Espeletia;* Berry & Calvo, 1989), and selfing for reproductive assurance (e.g. Melastomataceae; Manrique Valderrama *et al*., 2022). Furthermore, as bee species richness peaks at mid elevations in cloud forests (González & Engel, 2004), at higher elevations bumblebees and other insect pollinators such as dipterans and moths may become more predominant (Abrahamovich & Díaz, 2002; Wang *et al*., 2024; but the latter only has one data set from the Andean region) and vertebrate pollination is favoured (Dellinger *et al*., 2021b, 2023).

In addition, the tropical Andes also stands out for having a group of vertebrate pollinators that have notably radiated in this region: the hummingbirds (40% of all described species occur in the Andes; McGuire *et al*., 2014). Although hummingbirds occur from Alaska and Canada to Patagonia, clades unique to the region such as Coquettes and Brilliants diversified with the uplift of the Andes, while others such as Mangoes, Emeralds, Hermits and Bee hummingbirds colonised different areas or originated after Andean orogeny (McGuire *et al*., 2014). Such taxonomic richness is also expressed as a remarkable functional diversity, where key differences in morphology and behaviour have resulted in the exploitation of a broader spectrum of niches in comparison with other regions where hummingbirds are extant (Sonne *et al*., 2016). For instance, the hummingbird species with the largest and the smallest bills can be found coexisting in the same assemblage in the Colombian Andes (Rico‐Guevara, 2008). Additionally, tropical Andean hummingbirds have smaller ranges and higher levels of endemism (Sonne *et al*., 2022a), and have even colonised ecosystems at high elevations (Stiles, 2008) with adaptations to harsh mountain conditions. One example of such adaptations is adjusted haemoglobin concentrations in response to decreased oxygen availability (Williamson *et al*., 2023). Throughout the complex evolutionary history of hummingbirds as pollinators (Barreto *et al*., 2024), variation in key traits such as bill shape and size, body mass, and even feet for clinging while drinking nectar (Colwell *et al*., 2023), has fostered trait‐matching and concomitant size and shape variation with floral morphology (Rico‐Guevara *et al*., 2021). This is classically explained with the example of the long bill of the sword‐billed hummingbird (*Ensifera ensifera*) matching the long corolla tubes of the flowers it pollinates (including species in the genera *Passiflora*, *Brugmansia*, and *Aetanthus*), but bill length varies throughout this hummingbird's range according to the richness of visited plant species. In these geographic mosaics of local adaptation the quality and probability of interactions are driven by the presence or absence of different species in plant–pollinator communities, creating specialised interactions that can also be evolutionarily labile (Abrahamczyk *et al*., 2014; Soteras *et al*., 2018).

In general, the high spatial variation in community composition and environmental contexts has led to differences in the likelihood, type, and effectiveness of plant–pollinator interactions, even at very small spatial scales (Vélez‐Mora, Trigueros‐Alatorre & Quintana‐Ascencio, 2021). For example, populations of evening primrose plants (*Oenothera epilobiifolia*) in the Venezuelan Andes exhibit lower nectar quality at a higher (4450 m a.s.l.) than lower elevation (3600 m a.s.l.) site and had no recorded visits from pollinators even though pollinators were present and visited other plants. Given that the total energy offered by these flowers was similar between these sites due to floral aggregation at the high‐elevation site, the lack of pollinator visits to *O. epilobiifolia* at higher elevation may be due to the low reward per flower visit not offsetting the cost of foraging. Additionally, although plants exhibited similar capacities for self‐compatibility at both elevations, pollinator visitation at lower elevations resulted in increased seed production (Rodríguez‐Sánchez *et al*., 2024).

Furthermore, since the probability, quantity and quality of interactions also depend on the temporal match between plant and pollinator, interaction networks are influenced by plant phenology and the movement of pollinators within or across habitat patches (Olesen *et al*., 2011; Guzmán, Chamberlain & Elle, 2021). In the tropical Andes, rainfall, rather than seasonal temperature changes, largely determines flowering pulses (e.g. Franco‐Saldarriaga & Bonilla‐Gómez, 2020). Consequently, the timing of flowering follows complex patterns that are variable across dry or wet years, which are determined by global and regional climatic variability (Poveda *et al*., 2020; Arias *et al*., 2021). In addition, flowering within and between species is not always synchronous, allowing plant communities at the same site to sustain assemblages of pollinators throughout the year (Pelayo *et al*., 2019) or pollinators to move between sites to exploit differences in plant phenology, for example across elevations (Gutiérrez, Rojas‐Nossa & Stiles, 2004).

The emerging picture is that plant–pollinator networks in tropical Andean mountains are highly variable at fine spatiotemporal scales, as a response to species turnover and patterns of endemism and diversity, differences in environmental variables across sites, and changing climatic conditions within and across years (Cuartas‐Hernández & Medel, 2015; Restrepo Correa *et al*., 2016; Rodríguez‐Sánchez *et al*., 2024). A better understanding of this complexity is necessary to understand the diversity, evolution, and vulnerability of plant and pollinator communities (Burkle & Alarcón, 2011) as well as their use in local human activities and value to people.

## II. KNOWLEDGE GAPS

To assess the current knowledge gaps about pollination ecology in the tropical mountain ecosystems of the Andes, we conducted a systematic literature search with a standardised protocol and set of key words (Pullin & Stewart, 2006; Page *et al*., 2021; see Appendix S2 and Fig. S2). We searched for published research articles that contained in the title, abstract, or key words, the words ‘pollen’ or ‘pollin*’ and ‘Andes’, ‘Andean’, ‘South Americ*’ or any Andean country as an indication of location (see Appendix S2 for search string). Given that Spanish is the main language of the Andean region and it is used for scientific publications on this region, we designed a bilingual search (Amano *et al*., 2021; Zenni *et al*., 2023) that (*i*) employed search terms both in English and Spanish (see Appendix S2) and (*ii*) used two databases: *Web of Science* as the global database of indexed research, and *Scielo*, which contains collections of local biodiversity and ecology journals in Latin America. We conducted this search on 28 December 2023.

We automatically excluded *Web of Science* categories related to analyses of fossil pollen (and unrelated topics, see Appendix S2) and removed duplicates. This resulted in 3109 publications, and we added 55 relevant articles that were not located by the automated search (Pullin & Stewart, 2006; Table S1). We checked all publications by reading titles and abstracts. During this stage, we further excluded studies that were conducted outside the Andean region, not original research (e.g. reviews), unrelated to pollination ecology or not directly about the plant–pollinator interaction (e.g. descriptions of pollen grain structures, phylogenetic reconstructions that did not consider interactions, and distribution models or descriptions of single species). For the remaining 1010 publications that were included in downstream analyses, we recorded country (or countries), elevation(s), ecosystem(s) type where the study was performed, year of publication, journal, language, taxonomic scope, and if the study included applied research (e.g. honey production or agriculture) (Table S1). We used country information to define if studies were carried out in the tropical or temperate Andes, both, or at a continental scale. Although Chile and Argentina have territories north of the Tropic of Capricorn, we defined any study in these countries as ‘temperate Andes’ for ease of classification of studies by country rather than searching for coordinates. Of the 1010 studies identified by our search procedure, we excluded 537 studies from for the temperate Andes from further analyses, giving a final total of 473 studies included in our review. Finally, we identified the major themes that each publication addressed and the main methods used (Table S1, Appendix S2), allowing each study to have several themes and methods when they were covered. Common research themes were: (*i*) functional, genetic and species diversity; (*ii*) phenology; (*iii*) species interactions; and (*iv*) resilience and adaptability to change (Appendix S1).

**Fig. 3:**
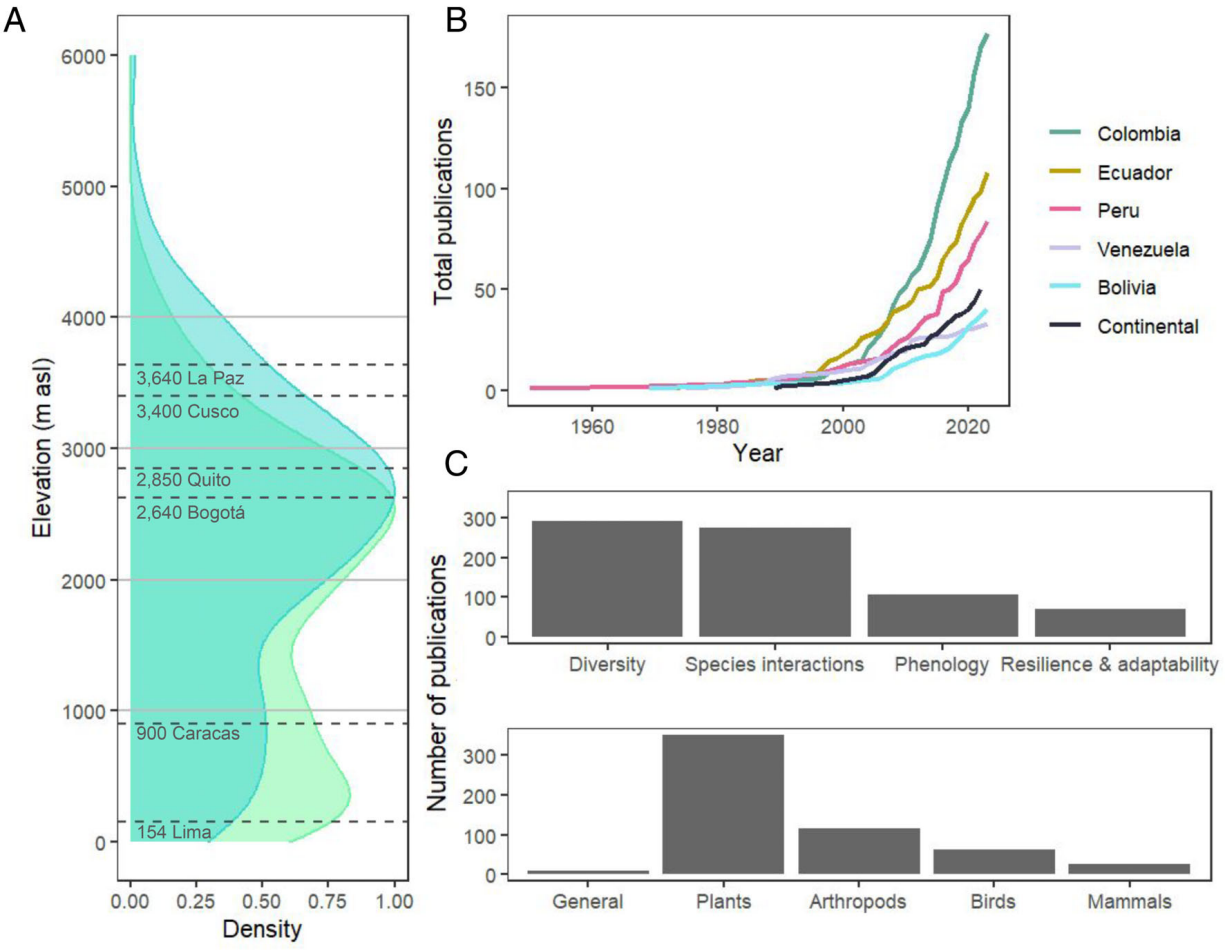
Geographic, temporal, thematic, and taxonomic distribution of research on pollination ecology in the tropical Andes. (A) Density of articles about pollination in the tropical Andes according to study site elevation in metres above sea level (m a.s.l.), with the green curve showing lower bounds and the blue curve showing the upper bounds of study locations. When studies were carried out at a single elevation, the lower and upper bounds are the same. Dashed lines indicate elevations of capital cities in tropical Andean countries, with the addition of Cusco, which was the capital of the Inka empire. Note that Caracas and Lima are not located in the Andes. The same plot by country is available in Fig. S3. (B) Accumulated total publications through time for each tropical Andean country. Studies with a continental approach (i.e. encompassing several countries and aiming for broad spatial coverage) are classified as ‘continental’. (C) Number of publications according to major research themes (top panel) and taxonomic groups (bottom panel). ‘Diversity’ refers to functional, genetic and species diversity. Studies that were not focused on a single group but rather studied pollination as a whole are classified as ‘General’.

**Fig. 4:**
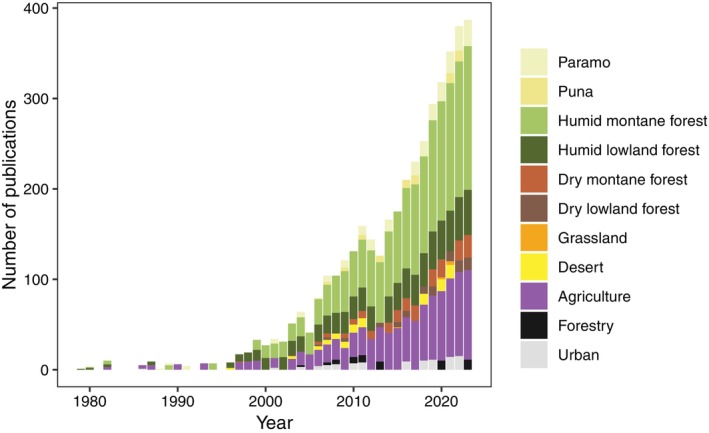
Natural and anthropogenic ecosystem types where research on pollination ecology has been conducted in the tropical Andes. Ecosystem types were extracted from publications that described the ecosystem where they were carried out (359 publications) and are classified as shown in the legend, based on the map of South America ecosystems provided by The Nature Conservancy (2008) (see Table S2) and adding three anthropogenic categories: agriculture (croplands and pastures), forestry and urban areas. We removed one very early study (1950, in agricultural ecosystem type in Peru) to ease visualisation. See Fig. S4 for plots by country.

Of the 1010 articles identified by our search, 414 (41%) were studies conducted in tropical regions (defined as in Venezuela, Colombia, Ecuador, Peru or Bolivia), 537 (53%) in temperate regions (defined as in Chile or Argentina), 9 (0.9%) encompassed both regions, and 50 (5%) were carried out at continental scales. Within the tropics, 18% of studies that reported elevation were located at low (below 1000 m a.s.l.), 11% at mid (1000–2000 m a.s.l.), 24% at higher mid (2000–3000 m a.s.l.), 11% at high (3000–4000 m a.s.l.), and 2% at very high (over 4000 m a.s.l.) elevations, whereas the remaining 35% covered elevational gradients encompassing more than one of these categories (Figs 3A and S3). The fact that a large proportion of studies have been carried out at higher elevations may reflect an Andean history of large human settlements in these areas, with three capital cities being located over 2500 m a.s.l. (Bogotá, Quito and La Paz). Most publications about pollination ecology in the region have focused on humid montane forests (37% of articles that described their study‐site ecosystems), with research in dry or alpine ecosystems increasing more recently (Figs 4 and S4). For anthropogenic habitats, most research has been done in croplands and pastures (23%) compared to urban (4%) and forestry areas (3%, Fig. 4). The number of studies has increased exponentially, notably in Colombia (a total of 174 published studies), followed by Ecuador (107) and Peru (83). There were fewer studies in Bolivia (40) and Venezuela (33) (Fig. 3B). Almost one quarter of published articles (22%) were written in Spanish, highlighting the importance of including local languages to gain a more complete picture of relevant scientific output.

Greater emphasis has been given to describing functional, genetic and species diversity, together with species interactions, whereas a focus on phenology or on the resilience and adaptation of pollination to change was less common (Fig. 3C). Of the published work we assessed, 5% focused on honey production and 23% on agriculture. Only 16 (3%) articles used local knowledge as part of their research. Most published work investigated pollination by using plants as the focal taxonomic group (62%; Fig. 3C), with plant chemical ecology (volatiles, 5% of total studies on plants), genomics (4%), species distribution models (SDMs, 2%) and network analyses (1%) receiving less attention within plant research in this field. There were fewer studies on the pollinators themselves (36% in total, with hymenopterans and birds being the most studied groups, with 82 and 61 articles, respectively; Fig. 3C). Particularly few studies have investigated methods of animal movement (3% of studies on animals), or used behavioural experiments (5%), genomics (2%), population genetics (0.5%), and SDMs (5%). It may be the case that our search terms, which focused solely on pollination, excluded publications on pollinating animals that did not explicitly consider their interaction with plant reproduction or make a direct link to pollination biology and ecology. However, we note that this is symptomatic of research ignoring the consequences of animal dispersal, seasonality, behaviour, and evolution on their role as pollinators, as well as the influences of plants on these animals. This represents a significant gap in animal research for the tropical Andes, and more attention must be given to studying plant–pollinator interactions from the pollinator's perspective. There is also a lack of publications that are not focused on taxonomic groups but take a holistic approach to characterising pollination at the community or ecosystem level (only 8 articles, 1% of total).

In the following subsections we present an overview of the scientific literature on pollination ecology in the tropical Andes, identifying knowledge gaps and summarising results from the perspective of the four major themes: functional, genetic and species diversity; phenology; species interactions; and resilience and adaptability to change.

### (1) Functional, genetic and species diversity

Due to the high species richness of the tropical Andes, it is unsurprising that research on pollination in this region has mainly focused on describing diversity (289 studies, 61%). By comparison, studies on pollination ecology in Chilean mediterranean ecosystems of the temperate Andes are available for less than 8% of plant species (Medel, González‐Browne & Fontúrbel, 2018), a number that could potentially be even lower for the tropical Andes, since this region is highly biodiverse. Baseline information on pollination in the tropical Andes is still lacking even for economically important crops such as cacao and avocado (Dymond *et al*., 2021; Vansynghel *et al*., 2022). Continued efforts revealing the variation in forms and function of pollination in this region will undoubtedly enrich our understanding about the nature of interactions between plants and their pollinators, as well as reveal patterns in their diversity.

**Fig. 5:**
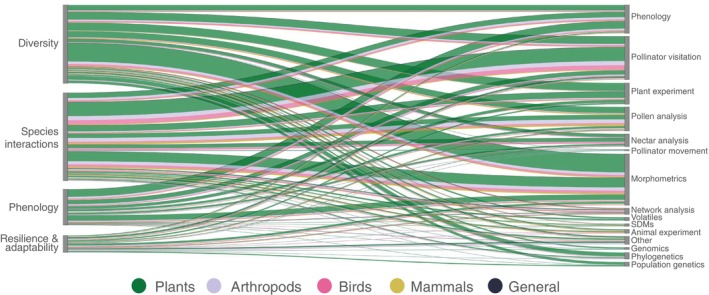
Relationships among major research themes, taxonomic groups and main methods used in the scientific literature on pollination ecology in the tropical Andes. Thickness of lines is proportional to the number of studies, with a line being drawn each time a major theme (left) or method (right) is included in a study, so several lines can be drawn if a study has more than one theme or method. Colours indicate taxonomic groups: plants (green), arthropods (lilac), birds (pink), mammals (yellow), and a general (black) approach to pollination (i.e. not focused on taxonomic groups). ‘Diversity’ refers to functional, genetic and species diversity; SDM = species distribution model; ‘Morphometrics’ and ‘Phenology’ include plant and pollinator morphometrics and phenology, respectively; ‘Other’ includes several other methods (Table S1). A histogram of studies by method is available in Fig. S5.

The most common method used to assess functional diversity has been through measuring morphological traits (266 studies, 56%), mostly for plants but also for pollinators (Figs 5 and S5). Key traits that influence pollination have been compared within clades and across groups of pollinators to study adaptation to certain interaction partners (Filipowicz & Renner, 2012; Muchhala & Thomson, 2010) and environmental contexts (Cuartas‐Hernández *et al*., 2019; Rodríguez‐Sánchez *et al*., 2024), including interspecific competition (Muchhala & Potts, 2007). Morphological traits that are key for pollination are also subject to selection (Lagomarsino *et al*., 2016), and variation in key traits can result in reduced niche overlap, thus explaining the occurrence of closely related sympatric species with differing reproductive strategies (Moreno‐Betancur & Cuartas‐Hernández, 2022) or highly specialised feeding habits (Mauck & Burns, 2009). The reconstruction of ancestral states of traits has been frequently used to explain shifts in plant pollination syndromes and infer visiting pollinators (Weigend & Gottschling, 2006; Lagomarsino *et al*., 2017), and this has stimulated research in taxonomy and systematics (52 studies, 11%). Less attention has been given to uncovering the basis of phenotypic variation or quantifying gene flow and population structure (15 and 29 studies, 3 and 6%, respectively). Plants have been the main focus of genetic analyses (87 studies, 18% of total), with arthropods (13, 3%) and birds (4, 1%) included in genetic studies much less frequently and mammals not at all (0 studies).

The special focus on plants, followed by hymenopterans and birds as pollinators (Fig. 3C), has led to a taxonomic bias that excludes some pollination systems altogether or underestimates the number of possible pollinators. Such bias neglects less‐conspicuous taxonomic groups, traditionally dismissed as unrelated to pollination, or third‐party species that may influence pollination interactions. Nocturnal pollination, for example, is typically understudied due to the increased difficulty of fieldwork at night, although some nocturnal pollinators such as bats (23 studies, 5%) and moths (2 studies, 0.4%) are known to be very important in this region, as well as globally (Muchhala & Potts, 2007; Buxton *et al*., 2022; González‐Gutiérrez *et al*., 2022). Moreover, studies that challenge previous research biases on pollinators may reveal groups of pollinators that were previously thought to not have such a role, such as fireflies visiting flowers of endemic paramo plants (Ladino Peñuela, Botero & Lima da Silveira, 2022). In other parts of the world, surprising pollinators have also been described, such as snails in India being important pollinators of morning glories (*Volvulopsis nummularium*) during rainy days when bees are not active (Sarma *et al*., 2007). By contrast, occasional pollinators may have limited influence on plant reproduction, but recognising their role in pollen transfer can broaden our knowledge about pollination strategies and network complexity (Ollerton, 2024; Requier *et al*., 2024). This is the case for rodent pollination, for which we only found three studies from Ecuador (Cárdenas *et al*., 2017; Dellinger *et al*., 2019; Nivelo‐Villavicencio, Timbe & Astudillo, 2021) and one in Colombia (Matallana‐Puerto & Cardoso, 2022). Lastly, species with an essential but indirect relation to pollination interactions have also been ignored. For example, we found no studies relating microbes in plants or soil to pollination, despite the growing evidence supporting their influence on plant–pollinator interactions in other parts of the world (Barber & Soper Gorden, 2015; Russell & Ashman, 2019).

### (2) Species interactions

Plant–pollinator interactions in the tropical Andes have been studied mostly through observational research, with floral visitation detected visually by humans or cameras (197 studies, 42%) or inferred by collecting pollen grains from the bodies of captured animals (part of pollen analysis methods; 139 studies, 29%, Figs 5 and S5). By recording the occurrence and frequency of interactions, these methods have revealed key properties of community structure, environmental variation and unexpected species associations. Network analyses (27 studies, 6%) have been useful to detect key species roles, describe community structure (e.g. Ramírez‐Burbano *et al*., 2017, Manrique‐Garzón *et al*., 2025), and relate spatiotemporal change in structural properties to the stability of pollination communities (Tinoco *et al*., 2017; Sonne *et al*., 2022b). Nevertheless, there is evidence that pollination networks are highly variable even at very fine spatial scales (Pelayo *et al*., 2021), so compiling more networks with standardised protocols across the region will be useful to understand broader patterns and underlying mechanisms of network structure, providing insight to questions related to the diversity and evolution of pollination interactions at a macroecological scale (as has been done for hummingbird–plant interactions, see e.g. Maruyama *et al*., 2018; Dalsgaard *et al*., 2021).

However, a bipartite model of pollination as a mutualism omits other interactions that also influence the plant–pollinator interaction. Although understudied, there is evidence of competition modifying resource selection (Weinstein & Graham, 2016) and herbivory affecting pollinator visitation through damage to floral morphology and reward (Chautá *et al*., 2017) and emission of volatile compounds (Kessler, Halitschke & Poveda, 2011). A notable case of direct and indirect competition influencing pollinator visitation to flowers is nectar robbing (Hazlehurst & Karubian, 2016). Nectar robbers shape the structure of plant–pollinator networks and can reduce plant fitness as they pierce holes that change behaviours of legitimate pollinators (Kjonaas & Rengifo, 2006; González & Loiselle, 2016). Although nectar‐robbing is common in other regions, the tropical Andes has a high diversity of specialised flowerpiercers (Rojas‐Nossa, Sánchez & Navarro, 2016). Flowerpiercers are birds in the tanager family (Thraupidae) that have hooked bills (Mauck & Burns, 2009), which they use to hold the flower's corolla, pierce it with the mandible, and drink nectar without coming into contact with the flower's reproductive structures. A few studies have shown that some flowerpiercers can also be legitimate pollinators, lending insight into how the mutualism–antagonism spectrum can vary with behaviour and morphology and highlighting the importance of considering their role in pollination networks (Pelayo, Rengifo & Soriano, 2011; Cuta‐Pineda, Arias‐Sosa & Pelayo, 2021).

By contrast, we found no studies in the tropical Andes on how predation affects pollinators' behaviour (Gavini, Quintero & Tadey, 2018), or how mutualistic–antagonistic interactions with fungi and microbes influence key pollination traits (Russell & Ashman, 2019; Wang & Tang, 2022; O'Neill, Brody & Ricketts, 2023). A closer look at these smaller life forms will certainly improve our understanding of how tertiary partners influence pollination interactions, such as phoretic mites hitching rides on hummingbirds to visit flowers possibly competing with their animal hosts for floral nectar (López‐Orozco & Cañón‐Franco, 2013). Additionally, there is a research gap on the role played by exotic species of plants, pollinators and tertiary partners, with a few examples showing how invasive plants are already integrated into pollination networks (Mackin *et al*., 2021; Quijano‐Abril *et al*., 2021) and the predominance of honeybees (*Apis mellifera*) over native bees in degraded landscapes (Cely‐Santos & Philpott, 2019). Invasive species strongly influence communities of pollinators and plants, and this has been extensively studied in the temperate Andes (e.g. Aizen, Morales & Morales, 2008; Sanguinetti & Singer, 2014; Chalcoff *et al*., 2022), which serves as a catalyst for prioritising similar studies in the tropical Andes too.

### (3) Phenology

The complexity of phenological patterns of both plants and pollinators in the tropical Andes warrants more research. We found 104 (22%) studies related to this topic, but much more evidence is required to describe broad spatiotemporal patterns because phenology is expected to be species and context specific. Marked changes in flowering are probably driven by precipitation seasonality, but this is poorly documented (Aguirre *et al*., 2011). Moreover, there is evidence of high variation in pollination network structure even at very small spatial scales (Cuartas‐Hernández & Medel, 2015). Asynchronicity in flowering is possibly driven by the benefit of sustaining pollinator assemblages throughout the year (Pelayo *et al*., 2021) and reducing competition between closely related plant species (Moreno‐Betancur & Cuartas‐Hernández, 2022). Tropical plants exhibit great variability in phenological rhythms (Sakai, 2001), and there is a large knowledge gap regarding the genetic basis underlying this diversity and their plasticity in response to changing climatic conditions (Satake, Nagahama & Sasaki, 2022). Most efforts to describe phenology have concerned plant flowering (Fig. 5), with almost no information on the seasonal movements of pollinators. Overall, researchers should aim to replicate their studies across sites and temporal windows to describe general patterns, as has been suggested previously for other complex mountainous regions (Medel *et al*., 2018).

### (4) Resilience and adaptability to change

Publications related to resilience and the capacity for adaptation of pollination in the tropical Andes are the least frequent (69 studies, 15%; Fig. 3C), despite the urgent need of data for management and conservation initiatives. There are more publications in this theme that incorporate local knowledge in their research (3% of total publications *versus* 15% of those focused on resilience and adaptability to change), and a greater use of methods classified as ‘other’ that are useful in describing responses to disturbance (e.g. land cover analyses represent 20% of publications in this theme *versus* 3% of total publications). However, some methods that could provide relevant insights into how pollination interactions are affected by change in this region are underrepresented in the literature, for example pollinator movement (1% of publications within this theme), phylogenetic analyses (1%), genomics (1%), animal experiments (3%), and species distribution models (4%, Fig. 5).

As research on resilience and adaptability to change accumulates for the tropical Andes, evidence has shown that plants and pollinators are affected by land conversion, fragmentation and pesticide use (Gutiérrez‐Chacón *et al*., 2018; Tinoco, Santillán & Graham, 2018; Struelens, Mina & Dangles, 2021; Obregón *et al*., 2021), invasive species have become integrated into interaction networks (Mackin *et al*., 2021), key roles of endangered species are lost as they disappear from unprotected habitats (Ramírez‐Burbano *et al*., 2017), and that habitat fragmentation causes pollen limitation, genetic bottlenecks and altered population dynamics (López‐A., Bock & Bedoya, 2008; López *et al*., 2021). Additionally, some studies have demonstrated that predicted changes in future climate may lead to reduced available niches (e.g. González *et al*., 2021) and associated co‐extinction of interaction partners in plant–pollinator networks (Matallana‐Puerto *et al*., 2022; Sonne *et al*., 2022b). The variety of threats and their implications for pollination will be increasingly overwhelming as evidence accumulates, and yet research has failed to predict change and generate direct recommendations. The few exceptions have shown that improved predictions should assess spatial and temporal heterogeneity of interactions, since the rate of coextinctions is variable across ecological communities (Sonne *et al*., 2022b) and assessments of resilience are conditioned by the complexity that we recognise within interactions (Montesinos‐Navarro *et al*., 2017).

There have been only a few studies that have tested management strategies and identified tangible means for recovering plant–pollinator interactions in the tropical Andes. A study in western Colombia found that active restoration was more effective than natural regeneration in augmenting pollinator visitation and fruit production in an aroid plant (García‐Robledo, 2010). In the southern Andes of Ecuador, network metrics of plant–hummingbird interactions were used to select plant species that are key for restoration (Crespo *et al*., 2022). In rural settings in Colombia a study showed that artificial feeders for hummingbirds have the potential to increase connectivity between habitat patches (Ramírez‐Burbano *et al*., 2022), although the generalised use of feeders remains hotly debated and more conclusive evidence is needed (Echeverry‐Galvis *et al*., 2024). More research in anthropogenic landscapes could give insights into how pollination interactions have responded to change, but comparatively few studies are from urban areas or managed forests (4% and 3%, respectively, of the 359 articles that described studied ecosystem types). Overall, knowledge gaps remain substantial, and the urgent need for data to inform conservation and management actions demands more applied research and greater involvement of local communities.

## III. FUTURE PERSPECTIVES

**Fig. 6:**
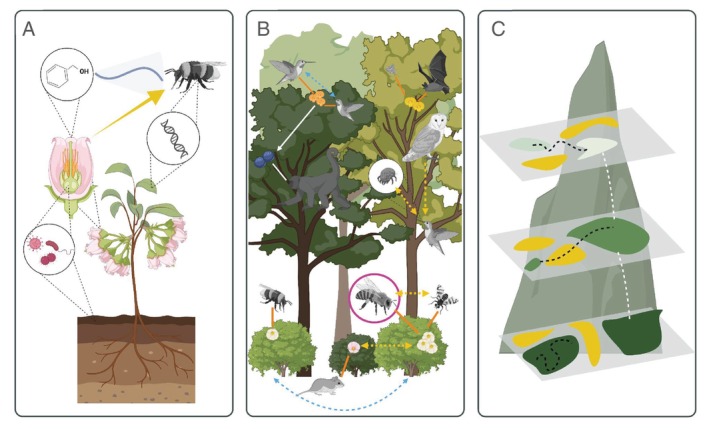
A cross‐scale approach to the study of pollination ecology. Pollination includes key players that range from the level of molecules, genes and microorganisms (A) to the web of interactions of communities (B) and landscapes across elevation gradients and geographic regions (C). (A) Circles highlight microscopic factors that can influence pollination, including volatile molecules that attract pollinators to flowers, microorganisms resident in the soil or plant organs, and the genes of both plant and pollinator partners. (B) Web of interactions at the community level, with orange lines showing floral visitors as potential pollinators, and white lines showing fruit development and seed dispersal. Dashed lines indicate additional biotic interactions within (blue) and between (yellow) species, such as direct competition (hummingbirds in territorial dispute), and indirect competition (fly and bee feeding on nectar, shrubs competing for light under the canopy), predation (owl hunting hummingbird), and interaction antagonism (phoretic mite on hummingbird competing for flower nectar). The honeybee is in a pink circle to depict invasive species. (C) Landscape configurations of habitats within and across elevational bands, which can also be approached at large continental scales. Colours indicate different habitat types, black dashed lines show movement within or between habitat patches and white dashed lines indicate movement across elevations. Note that illustrations are not to scale, and they represent functional groups rather than species. Figure created in BioRender (https://BioRender.com/o34w763).

The scale of organisation of the key players and processes involved in pollination ranges from genes and molecules to organs, organisms, populations, communities, ecosystems, landscapes and biogeographic regions. In this sense, research that embraces complexity and avoids excluding key abiotic or biotic factors will contribute most strongly to the field (Levine & Hart, 2020). A cross‐scale approach places pairwise interactions into a wider ecological and evolutionary context with population or community‐level studies (Fig. 6), improving predictions of change. Research on pollination ecology at a global scale has increasingly adopted a network‐based view (Knight *et al*., 2018) and plant–pollinator conservation has been encouraged to adopt a systems approach (Borchardt *et al*., 2021), providing the basis to recognise pollination as a vital process that underlies ecosystem structure and has deep implications for human livelihoods and wellbeing (Potts *et al*., 2016a).

Single research projects typically cannot convey complexity across all scales, but broadening awareness of the field's status and needs will enable research questions to be more impactful and interconnected. International and interdisciplinary research networks have the potential to (*i*) integrate relevant methodologies and standardised protocols for data collection, through shared knowledge and training, (*ii*) secure resources to support the longer‐term continuation of projects that have already started to flourish across the tropical Andes, and (*iii*) expand research to additional sites and subregions, especially those that have been underexplored.

In this section, we outline future research avenues that should be prioritised to advance the field of pollination ecology in the tropical Andes. We highlight the need to monitor spatiotemporal variation, conduct experiments focused on behaviour and ecophysiology, track movement of pollen and pollinators, model complex processes and stimulate participatory research that engages local human communities.

### (1) Monitoring spatiotemporal variation

Monitoring schemes collect data continuously, providing essential background data for longer term studies that can identify trends across time. Monitoring data collected in relation to pollination ecology may include, but are not limited to, occurrence and abundance of species and interactions, pollinator behaviour and movement, plant growth and physiology, abiotic conditions, etc. Monitoring is fundamental to capture the dynamic nature of pollination interactions (CaraDonna *et al*., 2017), since snapshots are insufficient to encompass the variability of the interaction and its ecosystemic role. Data collected by monitoring also serve as a baseline to assess change and suggest management goals. Spatial variation may be captured by forming collaboration networks that include various study sites, with standardised protocols to make comparisons possible. It is challenging to identify the most urgent monitoring needs, and monitoring programmes require investment of both funds and human resources. However, the cost of monitoring is likely to be much lower than the economic value of pollination services (Breeze *et al*., 2021). Ideally, monitoring protocols should be general enough that numerous research questions can use the collected data. A good strategy is to monitor several taxonomic groups rather than only a few, providing a community‐level approach that reveals aspects of system structure and stability. Additionally, monitoring tertiary partners outside the pollinating relationship will allow the study of multilayer networks with more than one interaction type (Dáttilo *et al*., 2016; Luna & Dáttilo, 2021), variation across time and space (Pilosof *et al*., 2017), and the influence of abiotic properties. Monitoring protocols can range from traditional surveys (O'Connor *et al*., 2019) to continuous detection of floral visitors using camera traps (van der Niet *et al*., 2022) or triggering systems (Rico‐Guevara & Mickley, 2017), schemes where citizen scientists help collect data (Birkin & Goulson, 2015), or more sophisticated techniques such as computer vision and artificial intelligence to identify interactions and describe behaviour (Ratnayake *et al*., 2023). The use of genetic tools may be particularly useful, with DNA barcoding and environmental DNA (eDNA) sampling and metabarcoding uncovering hidden diversity and interactions (Carrasco‐Puga *et al*., 2021; Marconi *et al*., 2022; Johnson *et al*., 2023) such as for microbiomes of plants and pollinators (Luna *et al*., 2023). With the possibility of constructing large data sets that should be maintained and curated through time, it is important to reflect not only on how data will be collected, but also consolidated, curated, stored and shared (following FAIR principles, see Wilkinson *et al*., 2016). Monitoring schemes may be inserted into national strategies (e.g. for Colombia, see Nates‐Parra, 2016) to ensure continuity, relevance and scope.

#### (a) Case study: collaborative network to monitor pollination in the high Andes of Venezuela

**Fig. 7:**
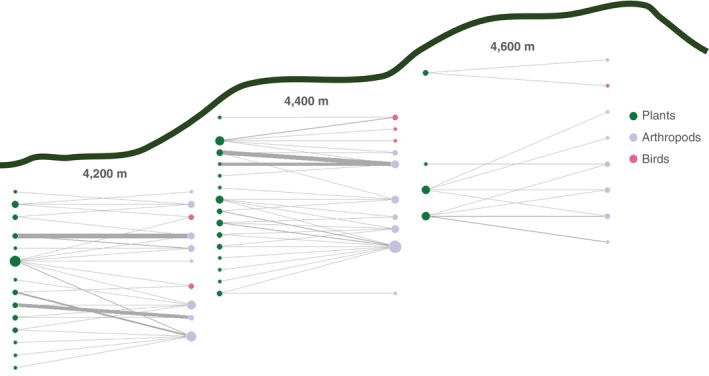
Variation of monitored interaction networks across elevations. Monitoring is part of the GLORIA‐Andes project in Piedras Blancas, Parque Nacional La Culata, Venezuela at 4200, 4400 and 4600 m above sea level (a.s.l.). All networks are highly specialised and have marked seasonal fluctuations, but the two lowest summits have similar species richness and structural indices compared to the highest site. Across sites, probabilities of interactions are linked to plant species occurrence, plant phenology and sampling effort. Nodes on the left side of networks indicate visited plants and nodes on right show pollinators, with colours indicating different taxonomic groups as shown in the legend. Thickness of connecting lines show frequency of visits and size of nodes represent degree centrality. Figure adapted from Pelayo *et al*. (2021).

The Global Observation Research Initiative in Alpine Environments (GLORIA) is a long‐term project that aims to assess the effects of global warming on montane ecosystems at a continental scale through standardised protocols that survey plant communities (Steinbauer *et al*., 2018). The creation of GLORIA‐Andes in South America was a key step to connect to this global initiative and generate monitoring data in a region where it is lacking (Inouye, 2020; Tovar *et al*., 2020). Venezuela has been part of the GLORIA‐Andes network since 2012, and has established seven vegetation monitoring summits located between 4200 and 4600 m a.s.l. Starting in 2018, a baseline for the annual phenological flowering pattern of summit plants and plant–pollinator networks was also established in the Piedras Blancas study area to provide mechanistic indicators to investigate how interactions that shape plant reproduction are responding to climate change (Fig. 7). Results thus far indicate that these high tropical alpine plant communities and their plant–pollinator networks could be particularly vulnerable to the loss of species in climate change scenarios, given their low species richness and functional redundancy, coupled with a high degree of specialisation and endemism (Pelayo *et al*., 2021). In accordance with the GLORIA protocol of repeating monitoring every 5 years, the Venezuelan sites were again surveyed for plant phenology and pollination interaction networks between 2023 and 2024, generating data that are currently under analysis. Continuation of long‐term monitoring schemes will enable robust assessments of community responses to climate change, but participation of other high tropical mountain locations within GLORIA is yet to take place. Joining such continent‐wide initiatives provides the potential for exchange of information such as new methods and local training.

### (2) Experiments focused on ecophysiology and behaviour

Experimental approaches can enable research to establish causal relationships that are relevant for pollination, with the potential for revealing complex direct and indirect effects of a variety of abiotic and biotic factors on behaviour, morphology and physiology, and, ultimately, the mechanisms that drive and sustain pollination, such as network stability and species fitness (e.g. Fründ *et al*., 2013; Biella *et al*., 2019). Experiments have primarily been carried out on plant reproduction (115 studies, 24%), focusing on the study of pollen limitation by assessing seed and fruit set. However, more detailed studies on physiology and behaviour that incorporate animals in experimental setups (only 14 studies, 3%) can also unveil morphological adaptations that influence pollinator attraction as well as pollen transfer, efficiency, and receptivity (Rengifo, Cornejo & Akirov, 2006; Muchhala & Potts, 2007; Muchhala & Thomson, 2010; Muchhala *et al*., 2014; Policha *et al*., 2016; Costa *et al*., 2023). Experimental research in the temperate Andes, for example, has described visual and olfactory cues that are key for pollinator attraction, vary geographically and have driven floral diversification (Schlumpberger & Raguso, 2008; Schlumpberger *et al*., 2009; Moré, Cocucci & Raguso, 2013; Moré *et al*., 2020).

In addition, we highlight that manipulating abiotic conditions to simulate future scenarios is important to anticipate responses and inform management strategies. For example, Guevara *et al*. (2023) controlled resource availability to test the effects of land use change on competition between hummingbirds. They found that forest conversion affects selection of the best artificial feeders in the presence of competitors, but that this trend changed across elevations and species assemblages. Similarly, González *et al*. (2022) increased temperatures in controlled conditions to study thermal thresholds that affect bumblebee physiology at different elevations. Their results showed that high mountain bumblebees could survive similar maximum temperatures as lower elevation bumblebees, suggesting that other drivers related to climate change, such as drought or competition, may be more important for future persistence. The use of elevational gradients as natural laboratories is a way to compare predicted responses in different environments [e.g. Tito, Vasconcelos & Feeley, 2020; Olsen *et al*. (2022) in Norway].

#### (a) Case study: using OTC‐based research to evaluate pollination in a warmer world

**Fig. 8:**
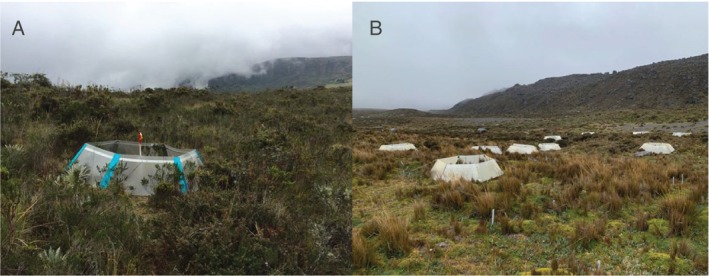
Experimental approach to simulate climate change with open‐top chambers (OTCs). OTCs set up in the Paramo Sumapaz in Colombia at 3500 m above sea level (a.s.l.) (A) and in the Paramo in the Antisana Volcano in Ecuador at 4580 m a.s.l. (B). Photographs by Eloisa Lasso.

The IPPEX network (International network of paramo and puna experimental warming sites) has pioneered a series of *in situ* warming experiments using open‐top chambers (OTCs) to simulate future warmer climates for high‐Andean mountain ecosystems. OTCs have polycarbonate walls hinged together into a hexagonal shape with an open top, warming the air inside the chamber through delayed escape of infrared radiation while also allowing airflow, rainfall, and movement of animals such as pollinators (Fig. 8). This experimental approach can be used to predict future climate change impacts on mountain biodiversity and ecosystem services, and to assess the ecological consequences of warming. The IPPEX network's collaborative, multi‐site strategy across the Andes also allows investigation of the spatial variability of responses of paramo and puna ecosystems to climate change.

A critical future focus for the IPPEX network through OTC‐based research is exploring how warming influences plant reproductive processes, including phenology, pollination, and fertility. Experiments conducted in Ecuador and Colombia using OTCs have consistently demonstrated gradual shifts in vegetation dynamics in response to warming (Duchicela *et al*., 2021; Lasso *et al*., 2021; Solarte *et al*., 2022). Preliminary findings from Ecuador indicate alterations in floral investment without corresponding phenological changes, while observations from Colombia suggest that warming may advance or delay flowering times, depending on the species, potentially disrupting the spatial and temporal patterns of pollinator interactions. OTC experiments can also explore how warming affects floral traits and displays, which in turn influence pollinator behaviour and ultimately impact reproductive success. Variations in precipitation, seasonality, elevation, vegetation, and land‐use history of the sites in the network are crucial for understanding broader patterns and pinpointing the conditions under which warming will exert the most significant impacts. The effects of warming on the phenology and reproduction of these unique ecosystems remain poorly understood, highlighting a significant knowledge gap that OTC‐based research in the tropical Andes is exceptionally positioned to fill.

### (3) Tracking movement of pollen and pollinators

Pollinator movement is informative of their foraging behaviour, seasonality, dispersal, and range shifts, and therefore is relevant for plant reproduction and population dynamics through patterns in floral visitation, pollen transfer and deposition, and ultimately gene flow. Methods to collect data on pollen movement include using fluorescent powder (Valdivia & González‐Gómez, 2006) and labelling pollen grains with quantum dots (Minnaar & Anderson, 2019), as well as inferring pollen flow from the analysis of plant genetic differentiation (Dellinger *et al*., 2022; Gamba & Muchhala, 2023). Pollinator movement can be detected and described from direct observations (Smith *et al*., 2008b), mark–recapture (García‐Robledo *et al*., 2004), estimating seasonal species distributions with citizen science data (Rueda‐Uribe *et al*., 2024a), analysing stable isotopes (Paxton *et al*., 2020), and animal tracking with telemetry (Hazlehurst & Karubian, 2018).

Animal telemetry is the method that directly measures an individual's trajectory, providing detail on pollinator movement. It can be applied over larger spatial and temporal scales compared to direct observation by humans or use of cameras, since transmitters are attached to animals and may communicate location data remotely. We found only one study related to pollination ecology that tracked animal movement with telemetry in the tropical Andes (Hazlehurst & Karubian, 2018), possibly because it is still difficult and expensive to track small species despite the relatively recent technological advances (Wilmers *et al*., 2015). A variety of technologies are available, with the choice depending on the research questions, available budget, and study species. The size of the study species is important because transmitters need to be as light as possible to avoid adverse effects on the animal's health or behaviour (Geen, Robinson & Baillie, 2019). Very light transmitters have been developed for deployment on small animals to generate near‐continuous tracking data. The largest tags incorporate Global Positioning System (GPS) transmitters to obtain information on an animal's coordinates. Lighter alternatives include geolocators, passive integrated transponders (PIT) and radio transmitters, but differences in their spatial and temporal resolution, storage capacity, detection distance, battery life and tag weight should all be carefully considered (Bridge *et al*., 2011). Outside the tropical Andes, research tracking pollinators has generally been focused on bees and bumblebees and has been used to determine space use and feeding preferences, particularly in croplands (e.g. Hagen, Wikelski & Kissling, 2011; Cavigliasso *et al*., 2020). More recently, telemetry studies in other regions have provided exciting new data that describe foraging activity and routes of pollinating bats (Goldshtein *et al*., 2020; Rivera‐Villanueva *et al*., 2024). Across taxonomic groups, tracking technologies are an essential toolbox that can enable the discovery of unknown movement patterns across different landscapes, improving inferences on the effects of climate and land use change, as well as enhancing related management strategies.

#### (a) Case study: fine‐scale and near‐continuous tracking of wild pollinators with radio telemetry

**Fig. 9:**
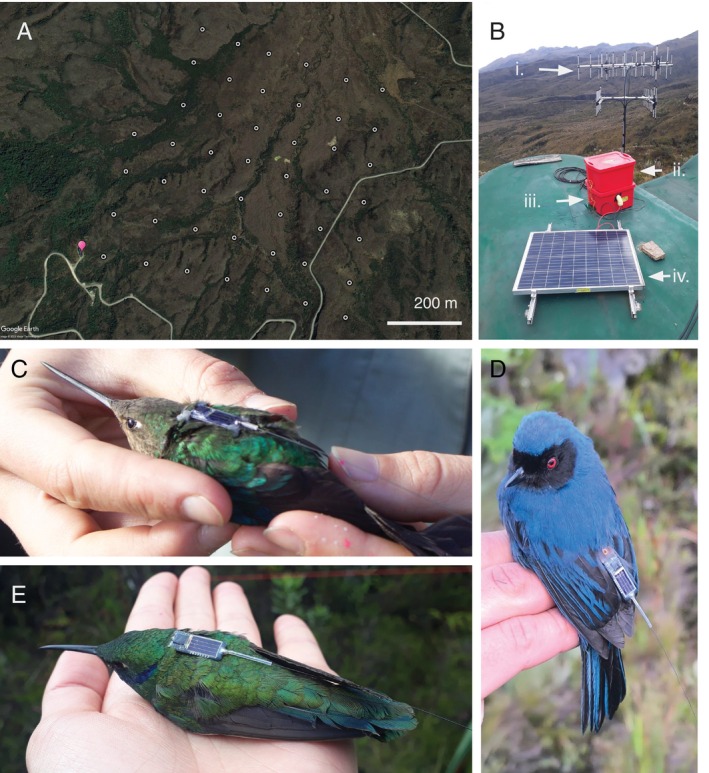
Automated radio telemetry system to track movement of pollinators in high‐Andean ecosystems. (A) Map of grid set up in the Valle de los Frailejones in Chingaza National Park, Colombia, with 46 receiving nodes (white points) and main antenna (pink flag). (B) Set‐up of the main antenna 10 m above the ground. Data are received from nodes in the valley by receiving yagi antennas (i) and downloaded using the Cellular Tracking Technologies (CTT) SensorStation (ii). Power is supplied to the system from a marine deep cycle battery (iii) that has additional charge from a solar panel (iv). (C–E) Birds with attached CTT LifeTags radio transmitters: (C) great sapphirewing (*Pterophanes cyanopterus*) with a hummingbird harness; (D) masked flowerpiercer (*Diglossa cyanea*) with a leg loop harness; and (E) sparkling violetear (*Colibri coruscans*) with glue‐on tag. Map made using *Google Earth* and photographs by Cristina Rueda‐Uribe (B, E), Manuela Lozano (C), and Pedro A. Camargo‐Martínez (D).

Automated radio telemetry systems (ARTS) currently provide the lightest tags for estimating continuous trajectories of animals. Tag weight starts at 0.06 g, allowing deployment on animals with a body mass over 1.2 g (maximum 5% of body mass). ARTS consist of arrays of receivers that store information about radio signal strength emitted by tags, which can be calibrated to estimate the distance of a tag to each receiver by timestamps. By overlapping data from three nodes or more, *x* and *y* coordinates of animal trajectories may be calculated. An ARTS grid to track pollinating hummingbirds and flowerpiercers has been set up in the Eastern Cordillera of Colombia in Chingaza National Natural Park, over 3150 m a.s.l. and covering elfin forest and paramo vegetation (Rueda‐Uribe *et al*., 2024b). It consists of 46 receiving nodes, which cover an area of approximately 0.7 km^2^ (Fig. 9). It has produced novel bird movement data at a fine spatiotemporal scale and may be used to investigate pollinator seasonality, home ranges, and association with vegetation types. In the future, other pollinators that may be tracked with this technology include bats, rodents, insects such as bumblebees, moths and butterflies.

### (4) Modelling complex processes

Conceptual and quantitative models help understand how multiple drivers are interconnected in determining the responses of complex systems and enable predictions of plant–pollinator communities under different scenarios. Pollination studies in the tropical Andes have used models to untangle driving factors of species radiations (Lagomarsino *et al*., 2016), identify the major variables that define species ranges (Matallana‐Puerto *et al*., 2022) and predict coextinction cascades (Sonne *et al*., 2022). Models can be tailored to the questions of interest. For example, Moré *et al*. (2021) used species distribution modelling to understand the environmental factors that influenced floral scent variation. Even though models may be restricted by temporal and spatial resolution, computing power, and the choice of scale, they have the capacity to test processes at different levels. For example, a spatially explicit individual‐based model to study the pollination ecology of the palm *Oenocarpus bataua* in northwestern Ecuador found that high co‐flowering density reduced pollen dispersal distance at a local scale, but the reverse pattern was true across the landscape (Díaz‐Martín *et al*., 2023). Despite such studies, key topics have not been investigated in the tropical Andes through modelling but have been addressed elsewhere. Examples include the effect of mating systems on genetic diversity (Marchelli, Smouse & Gallo, 2012), abundance of pollinators and pollination services across landscapes (Lonsdorf *et al*., 2009; Polce *et al*., 2013), and integration of possible effects of policy and market economies (Kremen *et al*., 2007). The development and application of models should aim to integrate major stakeholders, particularly if the goal is to anticipate losses of biodiversity or find management solutions, but such models have scarcely been used to predict change in the tropical Andes (with some exceptions, see e.g. Matallana‐Puerto *et al*., 2022; Sonne *et al*., 2022b) and there is an urgent need to formulate new models with nuanced context‐dependent drivers.

### (5) Engagement with local knowledge through participatory research

Participatory research engages local knowledge from the early stages of projects to ensure a more accurate assessment of needs, motivations and management opportunities. A real exchange between different types of knowledge (Groffman *et al*., 2010) can result in projects that are successful and sustainable in time. Participatory research can provide greater knowledge about the natural systems that are studied, and human presence and intervention in the landscape is also key to understanding current patterns of diversity. Unfortunately, we found that very few studies (16, 3%) used local knowledge in their research on pollination ecology in the tropical Andes, despite evidence of how the participation of local communities can help to identify pollinators (Beltrán‐Tolosa *et al*., 2020), recognise motivations to protect pollinators (Kolze *et al*., 2023), define the most viable management solutions (Bravo‐Monroy, Tzanopoulos & Potts, 2015; Struelens *et al*., 2021), and describe cultural processes that affect gene flow, diversity and evolution of staple crops (Parra‐Rondinel *et al*., 2021). People may be engaged in research projects on pollination through a variety of mechanisms, ranging from passive to active involvement. These include but are not limited to surveys, workshops, focus groups, citizen science and BioBlitzes, encounters between art and science, and science‐policy meetings. Much greater effort needs to be made by researchers to incorporate these methods into projects as a fundamental component rather than a secondary feature, using established frameworks that link ecological research with other disciplines such as social sciences and art (Adams *et al*., 2014). It is urgent for research on pollination ecology in the tropical Andes to integrate local communities because pollination is intrinsically linked to human systems through its influence on livelihoods and the economic value of pollination services (Olschewski *et al*., 2006; Ollerton, 2021), even in urban contexts (Silva *et al*., 2023).

#### (a) Case study: integrating specialised and local knowledge to restore pollination

**Fig. 10:**
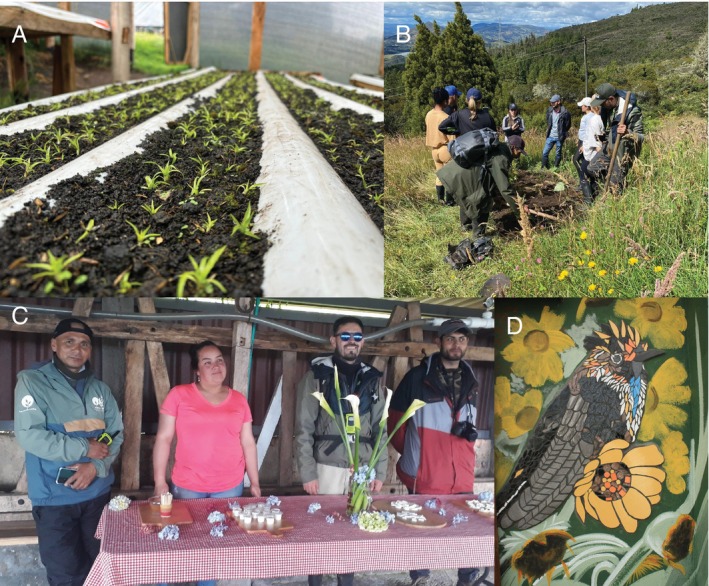
Examples of nature‐based solutions and research involving local communities. Activities carried out in El Zoque Nature Reserve in the Eastern Cordillera of Colombia include (A) a plant nursery with slow‐growing *Puya* sp. bromeliads of the paramo, experimenting with different growing techniques and employing local people; (B) active restoration efforts with community training; (C) farmers and nature guides sharing produce from goat milk as a productive alternative to dairy cows; and (D) an artistic mural made by young community leaders of a charismatic plant–pollinator interaction of the region (flowers of stem rosette *Espeletia grandiflora* and green‐bearded helmetcrest, *Oxypogon guerinii*). Photographs by Mauricio Restrepo (A, B) and Cristina Rueda‐Uribe (C, D).

Nature reserves have the potential to engage human communities to restore pollination services through the integration of specialised and local knowledge. One example is the private reserve El Zoque, located in the Eastern Cordillera of Colombia at 3000 m a.s.l. in a rural area not far from the capital Bogotá. It is dedicated to conserving remaining high‐Andean forest and paramo, using a strategy that involves local people in nature tourism, sustainable production and conservation (Fig. 10). Through nature tourism, the reserve generates funds and employment opportunities from the protection of natural ecosystems, provides a service of entertainment and connection to nature, and benefits from the knowledge of local guides about the landscape and its history. The reserve has also supported a shift from cow to goat dairy production to reduce localised impacts on plants and soils, and has a plant nursery for active restoration in adjacent areas that is managed by local young people and has the goal of increasing ecological connectivity for pollinators. By using observation data on plant–pollinator interactions, the reserve's nursery has selected keystone species to prioritise and establish in degraded areas to increase pollinator abundance. In addition, they have involved the local community in monitoring pollinators and educational activities related to pollination ecology, including the painting of a mural to depict pollinators and visited flowers. In this way, El Zoque has created opportunities for restoring and preserving fundamental ecological processes, renewed young peoples' interest in nature, and integrated specialised and local knowledge in management.

## IV. CONCLUSIONS

(1) Research on pollination ecology in the tropical Andes has increased exponentially, but there are thematic, taxonomic, methodological, geographic, and ecosystem‐level biases that lead to significant knowledge gaps. Publications on pollination ecology in this region have primarily described diversity and pairwise species interactions through methods of morphometrics and pollinator visitation, mostly focusing on plants and overlooking some groups of important pollinators as well as biotic and abiotic factors that influence pollination indirectly.
(2) It is concerning that local knowledge has been largely ignored in pollination research within the region, as engaging key stakeholders is essential for achieving meaningful impacts on policy and society.
(3) Moving beyond descriptive studies to understand the wider context of pollination and how natural communities can respond to change will enhance our ability to manage and protect pollination interactions amid ongoing environmental challenges.
(4) We propose a cross‐scale approach to capture the complexity of pollination, emphasising the importance of international and interdisciplinary research networks to drive progress of this field in the region. Collaborative networks can be particularly useful in integrating standardised data‐collection methods, supporting the continuation of existing projects, and expanding research to understudied locations.

## AUTHOR CONTRIBUTIONS

C. R.‐U.: Conceptualisation, Data curation, Formal analysis, Investigation, Methodology, Visualisation, Project Administration, Writing – original draft; A. C.: Conceptualisation, Data curation, Investigation, Methodology, Writing – review and editing; T. L. W: Data curation, Formal analysis, Investigation, Methodology, Visualisation, Writing – review and editing; E. L.: Supervision, Conceptualisation, Visualisation, Writing – original draft; R. C. P.: Supervision, Conceptualisation, Writing – original draft; L. M. M.‐G.: Visualisation, Writing – review and editing; M. C. M: Writing – review and editing; R. A.: Writing – review and editing; T.‐L. A.: Writing – review and editing; G. B.: Project Administration, Writing – review and editing; D. F. R. P. B: Writing – review and editing; P. A. C.‐M.: Writing – review and editing; M. A. E.‐G.: Supervision, Writing – review and editing; C. G.‐A.: Writing – review and editing; C. G.‐R: Writing – review and editing; L. T. L: Supervision, Writing – review and editing; K. K. S. L.: Supervision, Writing – review and editing; F. M.: Writing – review and editing; C. M.: Writing – review and editing; L. M.: Writing – review and editing; A. S. T. P.: Writing – review and editing; R. A. R: Supervision, Visualisation, Writing – review and editing; J. R.: Writing – review and editing; A. R.‐G.: Writing – review and editing; C. R.: Writing – review and editing; J. M. J. T: Project administration, Supervision, Writing – review and editing.

## Supporting information


**Appendix S1.** Current and future land cover projections.
**Appendix S2**. Literature search protocol.
**Fig. S1**. Projected areas of six land use and land cover classes in the tropical Andes region by country from 2020 to 2100 under five socioeconomic scenarios.
**Fig. S2**. PRISMA flow diagram for publications included in this review, following guidelines in Page *et al*. (2021).
**Fig. S3**. Density of articles about pollination in the tropical Andes by country, according to study‐site elevation in metres above sea level.
**Fig. S4**. Natural and anthropogenic ecosystem types for study sites where research on pollination ecology has been conducted in the tropical Andes, by country.
**Fig. S5**. Number of articles according to the main methods used.

**Table S1.** Database with reviewed articles and associated data.

**Table S2.** Ecosystem categories used in this study based on the classification of South American ecosystems published by The Nature Conservancy (2008).

## Data Availability

Data are available as online Supporting Information.
