# Supplementary material for: Pollination ecology in the tropical Andes: moving towards a cross‐scale approach
Source: Biol Rev Camb Philos Soc. 2025 Jul 15;100(6):2312–45. doi: 10.1111/brv.70049 (PMC12586307; doi:10.1111/brv.70049)
Supplement: Supplementary file 1 — Appendix S1. Current and future land cover projections. Appendix S2. Literature search protocol. Fig. S1. Projected areas of six land use and land cover classes in the tropical Andes region by country from 2020 to 2100 under five socioeconomic scenarios. Fig. S2. PRISMA flow diagram for publications included in this review, following guidelines in Page et al. (2021). Fig. S3. Density of articles about pollination in the tropical Andes by country, according to study‐site elevation in metres above sea level. Fig. S4. Natural and anthropogenic ecosystem types for study sites where research on pollination ecology has been conducted in the tropical Andes, by country. Fig. S5. Number of articles according to the main methods used. [file BRV-100-2312-s001.docx]

**Appendix S1. Current and future land cover projections**

We mapped current land cover projections for the region using 1 km spatial resolution from the HILDA+ version 2.0 data set (Winkler *et al*., 2025). Future land cover projections were generated by downscaling future land use and land cover change projections from LandSyMM (Rabin *et al*., 2020) at half degree resolution to approximately 1 km using the LandScaleR downscaling algorithm (Woodman *et al*., 2023; T.L. Woodman, B. Arendarczyk, K. Winkler, R.C. Henry, F. Eigenbrod, D.F.R.P. Burslem, P. Alexander & J.M.J. Travis, in preparation). HILDA+ land cover in 2020 was used as the baseline for downscaling. Land use and land cover change was downscaled separately for each country in the tropical Andes region using an f-value of 1.75, then cropped using a map of the Andes Mountains. The map for the Andes Mountains was extracted from the GMBA Mountain Inventory (Snethlage *et al*., 2022*a*,*b*). To this polygon, we added a 50 km buffer to include lowland areas that are still under Andean influence (for example inter-Andean valleys and foothills) and removed any areas in Chile or Argentina.

**Appendix S2. Literature search protocol**

(*A*) *Define relevant search terms*

English:

pollin* OR pollen AND Andes OR Andean OR South Americ* OR

Venezuel* OR Colombi* OR Ecuado* OR Peru* OR Bolivi* OR Chile* OR Argentin*

Search thread =

(TS=(pollin*) OR TS=(pollen)) AND (TS=(Andes) OR TS=(Andean) OR TS=(South Americ*) OR TS=(Venezuel*) OR TS=(Colombi*) OR TS=(Ecuado*) OR TS=(Peru*) OR TS=(Bolivi*) OR TS=(Chile*) OR TS=(Argentin*))

Español:

polin* OR polen AND Andes OR Andin* OR Suramérica OR Suramericano OR Sudamérica OR Sudamericano OR América del Sur OR

Venezuel* OR Venezolan* OR Colomb* OR Ecuado* OR Ecuatorian* OR Perú OR Peru* OR Bolivi* OR Bolivar* OR Chile* OR Argentin*

Search thread =

(TS=(polin*) OR TS=(polen)) AND (TS=(Andes) OR TS=(Andin*) OR TS=(Suramérica) OR TS=(Suramericano) OR TS=(Sudamérica) OR TS=(Sudamericano) OR TS=(América del Sur) OR TS=(Venezuel*) OR TS=(Venezolan*) OR TS=(Colomb*) OR TS=(Ecuado*) OR TS=(Ecuatorian*) OR TS=(Perú*) OR TS=(Peru*) OR TS=(Bolivi*) OR TS=(Bolivar*) OR TS=(Chile*) OR TS=(Argentin*))

Refined search:

Only research articles

Excluded topics:

Paleontology, meteorology atmospheric sciences, geology, archaeology, oceanography, pharmacology pharmacy, allergy, endocrinology metabolism, psychology, respiratory system, gastroenterology, hepatology, neurosciences neurology, general internal medicine, health care services, instruments instrumentation, pediatrics, dermatology, geriatrics gerontology, medical laboratory technology, otorhinolaryngology, radiology nuclear medicine medical imaging, international relations, research experimental medicine, dentistry oral surgery medicine, ophthalmology, cardiovascular system cardiology, fisheries, integrative complementary medicine, oncology, surgery, tropical medicine, urology nephrology, criminology penology, legal medicine, obstetrics gynecology, anesthesiology, architecture, astronomy astrophysics, family studies, hematology, medical ethics, medical informatics, optics, psychiatry, social work, sports sciences, telecommunications, theater, women’s studies, marine freshwater biology, geochemistry geophysics, mathematics, public environmental occupational health

(*B*) *Conduct literature search in databases* *(Web of Science as global and Scielo as regional) and download all results as an Excel file*

(*C*) *Perform general exclusion filters*

(1) Exclude all publications that are not original research (reviews, commentaries, editorial highlights, etc.)

(2) Exclude all publications that do not directly relate to pollination as an interaction

(3) Exclude all publications that are outside the Andean region

(*D*) *Tropical Andean exclusion filter*

Exclude publications from studies in temperate regions of Chile and Argentina

(*E*) *Extract information from selected references*

The following information was extracted from all references remaining after exclusion filters were applied:

- Title
- Authors
- Year
- Journal
- Main text language (EN = English, ES = Spanish)
- Location (country or continental)
- Region (tropical, tropical or temperate, temperate, or continental)
- Elevation (as given in the article or determined from coordinates given in the article)
- Ecosystem type [based on South America Ecosystems layer, published by The Nature Conservancy (2008), see Table S2]. 0 = absent; 1 = present.
  - Paramo
  - Puna
  - Humid montane/premontane forest
  - Humid lowland forest
  - Dry montane/premontane forest
  - Dry lowland forest
  - Deserts and xerophytic scrub
  - Natural grasslands
  - Crops and artificial pastures
  - Urban areas
  - Forestry
  - Other ecosystems
- Research theme (0 = absent; 1 = present)
  - Functional, genetic and species diversity: investigates some element of diversity, including species’ traits, genetic variation and structure, or species richness.
  - Phenology: investigating timing of biological events, for example flowering of plants, activity periods of pollinators, seasonal changes in occurrence and abundances of species.
  - Species interactions: focuses on the occurrence of interactions between species, including mutualistic interactions between plants and pollinators as well as antagonism (for example herbivory or nectar-robbing) and tertiary interactions related to pollination, including competition, parasitism, predation, etc. Research on species interactions can include, for example, recording the nature and frequency of interaction (e.g. floral visits), consequences of interaction (e.g. pollination efficiency) and interaction networks.
  - Resilience and adaptation to change: studies how pollination systems respond or adapt to environmental changes, for example habitat degradation and loss, introduction of exotic species, contamination and climate change; can also test or compare management and conservation strategies.
- Main methods (0 = absent; 1 = present)
  - Plant morphometrics
  - Plant phenology
  - Plant experiment
  - Pollinator visitation
  - Pollinator morphometrics
  - Pollinator phenology
  - Pollen analysis
  - Nectar analysis
  - Volatiles
  - Network analysis
  - Pollinator movement
  - Animal experiment
  - Population genetics
  - Phylogenetics
  - Genomics
  - Species distribution models
  - Other methods
- Taxonomic groups (0 = absent; 1 = present)
  - Unfocused
  - Plants
  - Bees and wasps
  - Beetles
  - Butterflies
  - Flies
  - Moths
  - Spiders
  - Ants
  - Arthropods (general)
  - Birds
  - Mammals
  - Other groups
- Use of local knowledge (0 = absent; 1 = present)
- Applied research (0 = absent; 1 = present)
  - Honey production
  - Agriculture

**
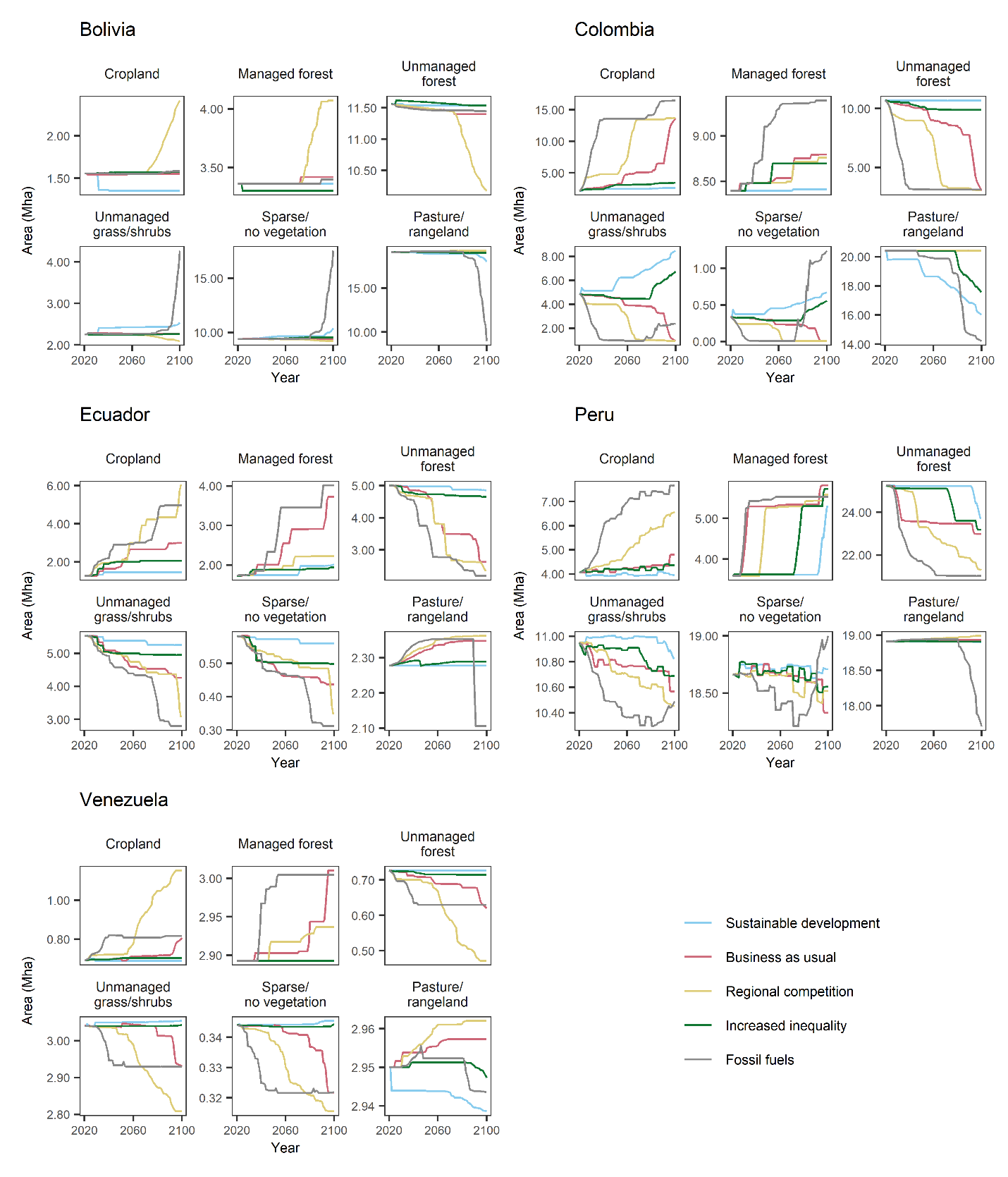
**

**Fig. S1.** Projected areas of six land use and land cover classes in the tropical Andes region by country from 2020 to 2100 under five socioeconomic scenarios (SSP1-RCP2.6, sustainable development; SSP2-RCP4.5, business as usual, SSP3-RCP7.0, regional competition; SSP4-RCP6.0, increased inequality, and SSP5-RCP8.5, fossil fuels). Note that ‘Unmanaged grass/shrubs’ is equivalent to ‘Unmanaged grass/shrublands’ in HILDA+. The map for the Andes Mountains was extracted from the GMBA Mountain Inventory (Snethlage *et al*., 2022*a*,*b*) and we added a 50 km buffer to include lowland areas that are still under Andean influence (e.g. inter-Andean valleys and foothills).


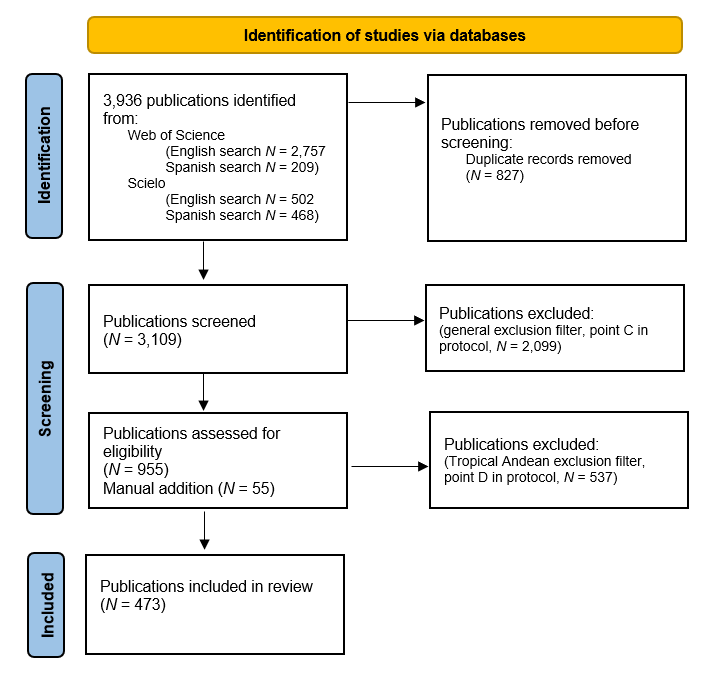


**Fig. S2.** PRISMA flow diagram for publications included in this review, following guidelines in Page *et al*. (2021).


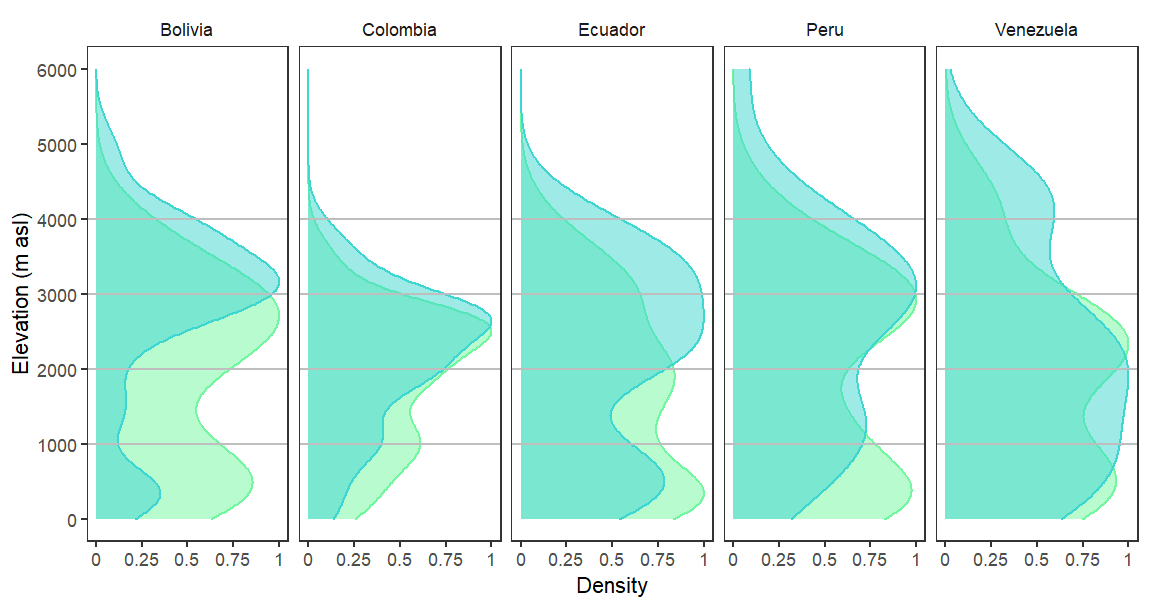


**Fig. S3.** Density of articles about pollination in the tropical Andes by country, according to study-site elevation in metres above sea level. The green curve shows lower bounds and the blue curve upper bounds of study locations. When studies were carried out at a single elevation, the lower and upper bounds are the same.


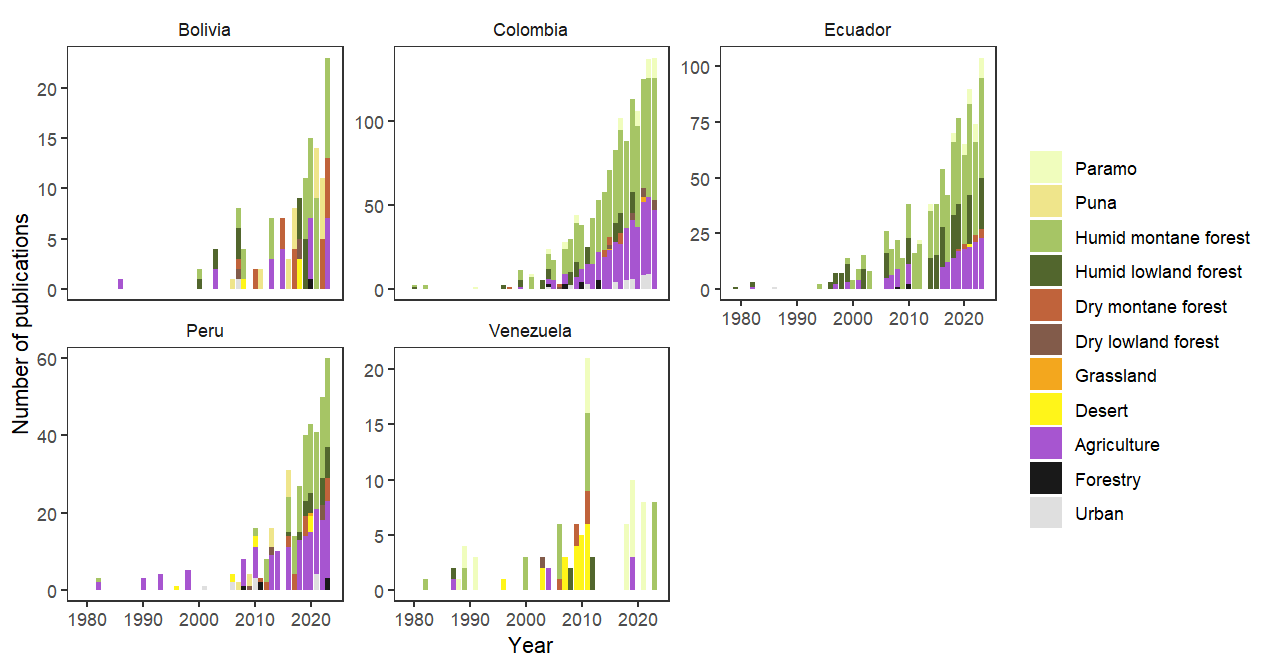


**Fig. S4.** Natural and anthropogenic ecosystem types for study sites where research on pollination ecology has been conducted in the tropical Andes, by country. Ecosystem types were extracted from publications that described the ecosystem where they were carried out (359 publications) and classified as shown in the legend, based on the map of South America ecosystems (provided by The Nature Conservancy, 2008; see Table S2) and adding three anthropogenic categories: agriculture (croplands and pastures), forestry and urban areas. We removed one very early study (1950, in agricultural ecosystem type in Peru) to ease visualisation.


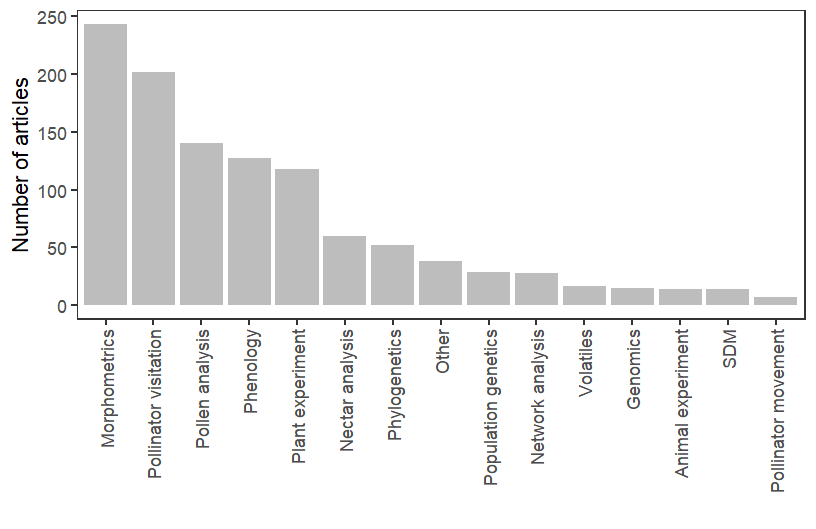


**Fig. S5.** Number of articles according to the main methods used. ‘Morphometrics’ and ‘Phenology’ include both plant and pollinator morphometrics and phenology, respectively; SDM = species distribution model; ‘Other’ includes several other methods (see Table S1).
